# Large-scale water collection of bioinspired cavity-microfibers

**DOI:** 10.1038/s41467-017-01157-4

**Published:** 2017-10-20

**Authors:** Ye Tian, Pingan Zhu, Xin Tang, Chunmei Zhou, Jianmei Wang, Tiantian Kong, Min Xu, Liqiu Wang

**Affiliations:** 1 Department of Mechanical Engineering, The University of Hong Kong, Hong Kong, China; 2 HKU-Zhejiang Institute of Research and Innovation (HKU-ZIRI), Hangzhou, Zhejiang 311300 China; 3 Center for Transport Phenomenon, Shandong Academy of Sciences, Jinan, Shandong 250103 China; 4 School of Chemical Engineering and Technology, Tianjin University, Tianjin, 300072 China; 5 Guangdong Key Laboratory for Biomedical Measurements and Ultrasound Imaging, Department of Biomedical Engineering, Health Sciences Center, Shenzhen University, Shenzhen, Guangdong 51800 China

## Abstract

Large-scale and high-efficient water collection of microfibers with long-term durability still remains challenging. Here we present well-controlled, bioinspired spindle-knot microfibers with cavity knots (named cavity-microfiber), precisely fabricated via a simple gas-in-water microfluidic method, to address this challenge. The cavity-microfiber is endowed with unique surface roughness, mechanical strength, and long-term durability due to the design of cavity as well as polymer composition, thus enabling an outstanding performance of water collection. The maximum water volume collected on a single knot is almost 495 times than that of the knot on the cavity-microfiber. Moreover, the spider-web-like networks assembled controllably by cavity-microfibers demonstrate excellent large-scale and high-efficient water collection. To maximize the water-collecting capacity, nodes/intersections should be designed on the topology of the network as many as possible. Our light-weighted yet tough, low-cost microfibers with high efficiency in directional water transportation offers promising opportunities for large-scale water collection in water-deficient areas.

## Abstract (web summary)

Spider-silk-mimicking microfibers often suffer from low efficiency and durability in water collection. Here, the authors fabricate robust microfibers with spindle cavity-knots and different topological fiber-networks with improved water-collecting performance

## Introduction

The spider silk^1, 2^ is well-known for its intriguing ability to collect water from humid air, and has thus inspired the design for materials of unique wettability. The water-wetted spider silk composes of periodic spindle-knots and joints with different surface roughness^3^. The unique structure of the natural microfiber enables a surface energy gradient, as well as a difference in Laplace pressure between the knots and joints^4^. Both result in the directional transport of water droplets towards the knots continuously. Guided by this insight, microfibers with polymer spindle knots have been fabricated to mimic the spider silks for water collection^5–11^. The functionalities of these microfibers depend crucially on their geometrical properties, such as the knot size and surface nanostructures.

The microfibers with spindle-knots can be fabricated by methods including electrospinning^12, 13^, dip-coating^14, 15^, and microfluidic approaches^16^. In the electrospinning approach, a viscous inner liquid and a less-viscous shell liquid are electrified coaxially, forming a hydrophobic fiber with hydrophilic knots^12, 13^. With the dip-coating, smooth microfibers are dipped into polymer solutions, and then droplets form along the fiber due to Rayleigh-plateau instability^14, 15^. Subsequently, these droplets are solidified to generate the knots. These two approaches have, however, limited control over the microstructure of the fabricated fiber, such as the separation between the knots and the size of the knots. Microfluidics enables good controllability of microscale jets and droplets^17^, and is thus capable of producing microfibers with precisely-tuned spindle-knot structures. With the microfluidic approach, a liquid jet encapsulating discrete core oil droplets is typically templated for a gel fiber^18^. After dehydration, the oil cores wrapped in a thin layer of gel fiber serve as knots. Thus, the spindle-knot fiber by microfluidics is usually uniform in the material composition, almost with no difference in surface roughness. Moreover, the oil drops evaporate over time and thus the knots deform gradually, compromising the long-term functionality. Furthermore, although the functions of a single fiber have been studied, the integrated collective performance of assembled spindle-fibers as topological networks has not been sufficiently demonstrated. Therefore, it is urgently demanding to fabricate durable, functional spider-silk-mimicking microfibers, and assemble these fibers into topological networks for large-scale water collection.

Here we fabricate microfibers with spindle cavity-knots that mimic the structure and surface roughness of the spider silk from composite hydrogels (named cavity-microfiber) by simple microfluidics, for assembled networks and large-scale water collection. The cavity-microfibers are templated from jet phase with gas bubbles, via cross-linking and drying. The knot size and distance between the knots are controlled by the flow rates of the jet phase and the pressure of the gas phase. The surface roughness is enabled by incorporating phase-separated polymers in the jet phase. Owing to the cavity design, the surfaces of the knot part are much rougher than the rest of the cavity-microfiber, enhancing the driving force of the directional water transport. Due to the robustness of the cavity-microfiber, the cavity knots maintain their shape and functions for cycles of water-collecting. Furthermore, we demonstrate the water-collecting efficiency of different topological fiber-networks made from the cavity-microfibers in bio-mimicking spider-web. We show that the structure of cavity-microfiber and its network topology dictate the water-collecting performance. Our facial and economic approach offers light-weighted, yet tough spindle cavity-knot microfibers with high efficiency of water collection. These cavity-microfibers are promising building blocks for spider-web-like networks for water treatment, drug delivery, tissue engineering and cell culture.

## Results

### Fabrication of cavity-microfiber via one-step microfluidic method

**Fig. 1:**
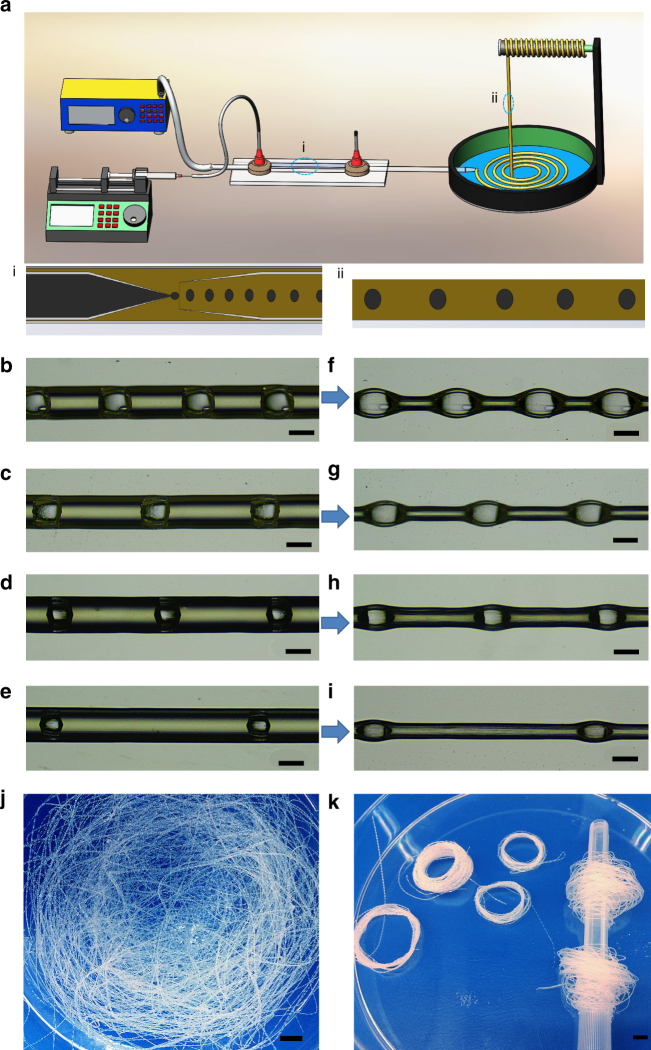
Schematic diagram showing the experimental set-up for the fabrication of cavity-microfibers. **a** Schematic diagram of the gas-in-water capillary-based microfluidic system. **b**–**e** Optical images of hydrated cavity-microfibers under different flow rates of continuous phase *Q*_*jet*_ and gas pressure of dispersed phase *P*_gas_(**b**) 0.6 mL h^−1^: 22.40 kPa; (**c**) 0.6 mL h^−1^: 20.96 kPa; (**d**) 1 mL h^−1^: 26.04 kPa; (**e**) 1 mL h^−1^: 24.07 kPa. **f**–**i** Optical images of dehydrated cavity-microfiber corresponding to (**b**–**e**). **j**–**k** The collected dehydrated cavity-microfibers in large quantities. Scale bars, 100 μm in (**b**–**i**) and 6 mm in (**j**–**k**)

We employed capillary-based microfluidic system (Fig. 1a) to generate simple gas-in-water jet templates. Alginate-based composite solution (ABC solution) was used as the continous jet phase; nitrogen or air was used as dispersed phase to generate bubbles^19–21^ under the shear of the continuous flow. Then the jet of ABC solution encapsulating uniform bubbles was solidified into hydrated microfiber with cavity knots (named hydrated cavity-microfiber, Fig. 1b–e). The aqueous jet was cross-linked instantly in the CaCl_2_ solution upon touching with the Ca^2+^ ions (Supplementary Fig. 1). Subsequently, the solidified hydrated cavity-microfiber was dehydrated at ambient temperature for >1 h (named dehydrated cavity-microfiber, Fig. 1f–i). The resultant dehydrated microfiber with spindle cavity-knots is light-weighted and has a density, 0.5929 g cm^−3^, which is much smaller than that of dehydrated microfibers without knots and with silicon-oil-knots, 1.1386 and 1.0728 g cm^−3^, respectively. The diameter of hydrated cavity-microfiber was controlled by the flow rate of continuous phase, *Q*_*jet*_.We varied *Q*_*jet*_ from 0.4 to 1.6 mL h^−1^, and the diameter of hydrated cavity-microfiber increases from 132.093 to 155.768 μm linearly (Supplementary Fig. 2). Under the constant *Q*_*jet*_, as the inlet pressure of the gas phase increased, the volume of the generated gas bubbles increased and the distance between the bubbles decreased (Supplementary Fig. 3a, b and Supplementary Fig. 4). Thus, the fiber diameter, the knot size and the distance between knots can be tuned conveniently. The production rate of cavity-microfibers is highly dependent on the flow rate of continuous phase *Q*_jet_. Varying *Q*_jet_ from 0.4 to 1.8 mL h^−1^ within 120 s, the length of cavity-microfiber fabricated increases from 12.8 to 52.24 cm linearly (Supplementary Fig. 5a). The linear relationship between fiber length and *Q*_jet_ holds over the fabrication time (Supplementary Fig. 5b). The production rate is 64–263 mm min^−1^ for a single channel (Supplementary Table 1). By using 10 channels simultaneously, ~ 158 m cavity-microfiber can be easily fabricated within 1 h under *Q*_jet_ = 1.8 mL h^−1^. The production cost of the fibers is estimated to be $0.157 for 100 m. The simple microfluidic approach enables the generation of such microfibers in large quantities, which are collected using a plastic rod (Fig. 1j, k) for storage and further use.

### Characterization of morphology of cavity-microfiber

**Fig. 2:**
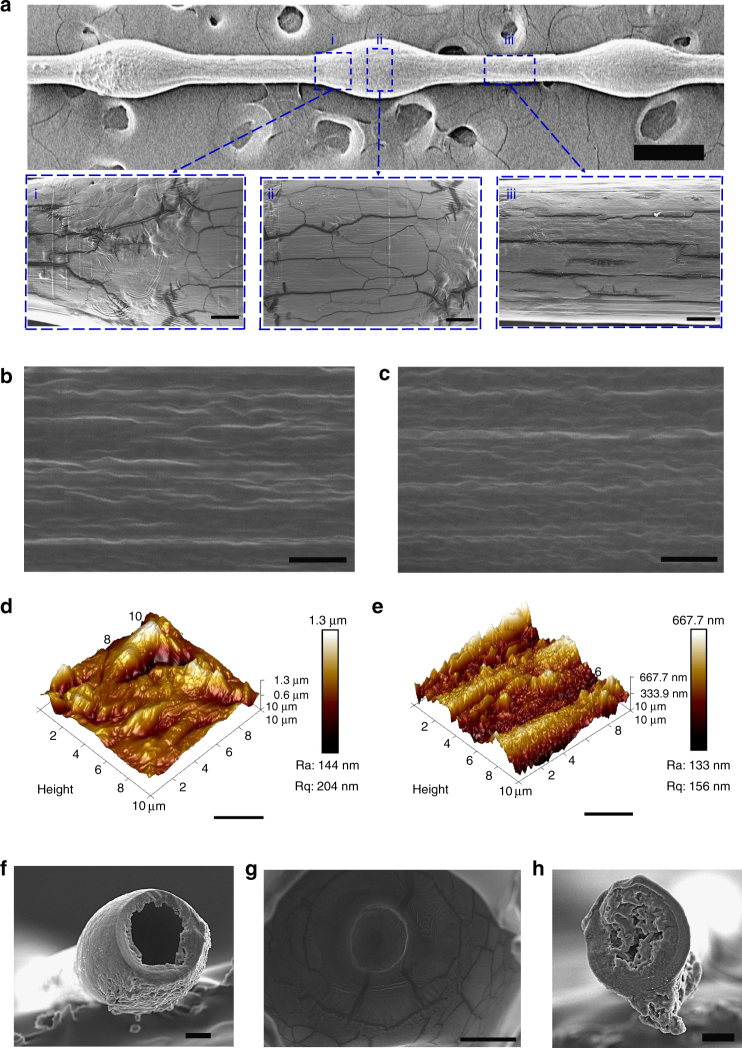
The morphology of cavity-microfibers. The SEM images showing (**a**) the dehydrated cavity-microfiber; (**b**) the nanostructures of knot part of cavity-microfiber; (**c**) the nanostructures of joint part of cavity-microfiber; AFM image showing roughness and nanostructures of (**d**) the knot part and (**e)** joint part of the cavity-microfiber; SEM images showing (**f**, **g**) the hollow cavity of the knot; (**h**) the solid cross section of the joint part for cavity-microfiber. Scale bars, 300 μm in (**a**), 30 μm in (**a**, i–iii), 2 μm in (**b**–**e)** and 50 μm in (**f**–**h)**

The resultant spindle cavity-knot microfibers from composite polymers (Fig. 2a) we fabricated have advantageous surface property, mechanical strength and durability. Intriguingly, the surface of the knot on the cavity-microfiber exihibits alligator cracking (Fig. 2a, i–ii), while that of the joint surface has transverse cracking (Fig. 2a, iii). After 30 measurements, the crack density^22^ of joint surface is estimated to be ~ 900 cracks per mm^2^, and the crack density of knot surface reaches up to ~ 2200–2734 cracks per mm^2^. The surface-roughness of the knot ~ 204 nm is much larger than that of the joint, ~ 156 nm, as shown in Fig. 2b–e. This feature resembles the surface morphology of a spider-silk fiber, and differs from fibers previously reported^16^, where the joint usually has rougher surface than the knot. This desirable surface morpology of the cavity-microfiber is asscoiated with the hollow knot and solid joint, as demonstrated in Fig. 2f–h. Before dehydration, the hydrated cavity-microfiber from ABC solution contains polyvinyl acohol (PVA) and polyethyelene glycol (PEG). During dehydration, the polymer concentation increases sharply due to water losing, and results in the phase seperation between PVA and PEG^23, 24^. The cavity-knot is dehydrated much faster than the solid joint, due to the significant large surface area. Therefore, the cavity-knot is freezed at the early time of phase seperation, the binary structures of which is featured by small domains, resulting in alligator cracking with high crack density; while the solid joint at the late stage of phase seperation is featured by large and continuous domains, leading to transverse cracking with low crack density. The enhanced roughness on the knot surface creates an elevated surface energy gradiant between the knot and joint, thus favoring the water transport towards the knot.

### Mechanical properties of cavity-microfiber

The mechanical strength such as stretching and bending abilities of cavity-microfiber from composite polymers are significantly improved. For instance, the composite cavity-microfiber, stretching to 300% of its the original length, which is 1.5 times more stretchable than the alginate fiber (Supplementary Fig. 6a, b). The maximum tensile stress of the cavity-microfiber, 2.238 MPa (Supplementary Fig. 7), is almost twice larger than that of alginate fiber, 1.31 MPa (Supplementary Fig. 8). In addition, the resultant cavity-microfiber can be easily spiraled (Supplementary Fig. 9a), folded (Supplementary Fig. 9b) and tied (Supplementary Fig. 9c), even assembled into 3D scaffolds (Supplementary Fig. 9d), demonstrating its excellent bending property.

### Investigation of water collection ability of single cavity-microfiber

**Fig. 3:**
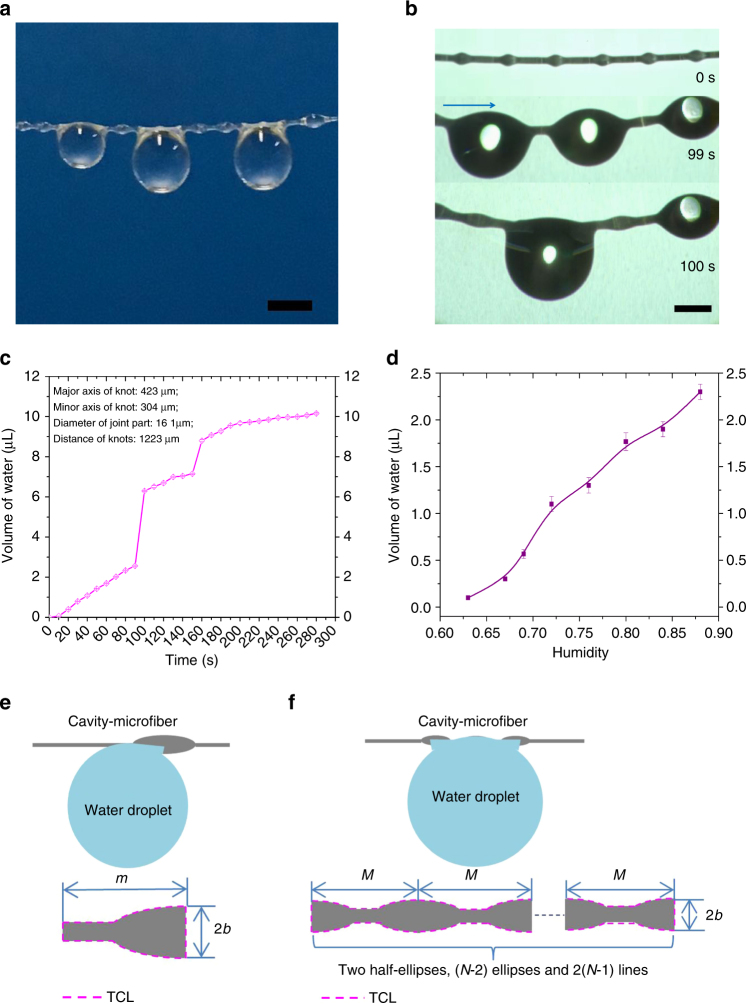
Water collection of single dehydrated cavity-microfiber. **a** The optical image of water droplets collected on a single dehydrated cavity-microfiber (fog flow: 0.408 mL min^−1^). **b** The water droplet collected on random knots move towards a domain knot (fog flow: 0.408 mL min^−1^). **c** The relationship between the time and the volume of water droplet collected (fog flow: 0.408 mL min^−1^). **d** The relationship between the humidity and the volume of water collected under airtight environment with no air convection. **e** The schematic diagram of TCL for water droplet detaching from one spindle knot of cavity-microfiber. **f** The schematic diagram of TCL for water droplet detaching from *N* spindle knots of cavity-microfiber. All error bars in (**c**) and (**d**) indicate the standard deviations over five independent measurements. Scale bars, 2 mm in (**a**) and 1 mm in (**b**)

To characterize the water-collecting ability of the fabricated cavity-microfiber, we first investigate a typical single cavity-microfiber, interacting with water in an artifical fog flow of 0.408 mL min^−1^ (Fig. 3a). At first, tiny water droplets were absorbed by the hydrogel cavity-microfiber, resulting in the slight swell of cavity-microfiber (Supplementary Fig. 10a, b). Then tiny water droplets condensed on the cavity-microfiber at random locations. The water droplets grew bigger due to the continuous condensation and moved toward the spindle knot directionally (Supplementary Fig. 11 and Supplementary Movie 1) to form a large water droplet, due to the difference in the surface energy^4, 25, 26^ (Supplementary Fig. 12 and Supplementary Note 1) and Laplace pressure^4, 13, 27^ (Supplementary Fig. 13a, b and Supplementary Note 2) between the knot and joint. A knot with a large difference of Laplace pressure or a large surface energy can be the domain knot among others. The water droplets collected on the knots moved towards the domain knot, thus an even larger droplet was collected (Fig. 3b, Supplementary Fig. 14, Supplementary Note 3 and Supplementary Movie 2). We monitored the water collected on a domain knot over time (Supplementary Fig. 15). The water volume increased linearly with time, except two sharp increases, indicating the transporting and merging of water droplets on the domain knots (Fig. 3c). The maximum volume of water droplet collected by the domain knot was 10.13 μL (Supplementary Fig. 16a), almost 495 times than that of the cavity knot itself, 0.0205 μL. The water volume collected on single domain knot of the cavity-microfiber is at least 40% higher than fibers previously reported^8, 10, 11^ when collecting time is longer than 150 s (Supplementary Table 2), although we note that the experimental conditions are not strictly the same by different groups. For a single spindle-cavity-knot microfiber of length 7.6 cm, the water volume collected in 600 s increases from 0.1 to 2.2641 μL, as the humidity increases from 63 to 88% in the airtight container (Fig. 3d).

The maximum water capacity that collected by single cavity-microfiber is determined by a balance between the water gravity and the adhesion to the fiber. As the water droplets became sufficiently large on the knots, they detached and fell off. The length of the three-phase contact line (TCL) *L* can be expressed as *L* = 2*m* + *πb* for a single knot from which a water droplet detached (Fig. 3e and Supplementary Note 4), where *m* is the contact length between the fiber and water droplet, and *b* is the minor semi-axis of the spindle knot^11^. By balancing the adhesive and gravitational effects, we have the maximum volume that can be collected by a single knot as:

1 $${V_m} = \frac{{\gamma \cos \theta L}}{{\rho g}} = \frac{{\gamma \cos \theta }}{{\rho g}}\left( {2m + \pi b} \right),$$

where *γ*, *ρ* are the surface tension and density of water, *g* is the gravitational acceleration, and *θ* is the contact angle of the water droplet on the fiber^11^. In general, the water capacity of the cavity-microfiber can be enhanced by increasing the TCL. If the water droplet is collected on two or more identical neighboring knots, the TCL immediately increases. For multiple identical knots of number *N* ≥ 2, the length of TCL is expressed as (Fig. 3f):

2 $$L = 2\left( {N - 1} \right)M + 2b\left( {N\pi - \pi - {\rm{2}}N + 4} \right),$$

where *b* is the minor semi-axis of these spindle knots, *M* is the contact length between the fiber and water droplet. Therefore, we can evaluate the max volume of water droplet collected by single cavity-microfiber (Supplementary Fig. 16b and Supplementary Note 5):

3 $${{V_M} = \frac{{\gamma \cos \theta L}}{{\rho g}} = \frac{{2\gamma \cos \theta }}{{\rho g}}\left[ {(N - 1)M + b(N\pi - \pi - {\rm{2}}N + 4)} \right]}$$

### Durability of the cavity-microfiber

**Fig. 4:**
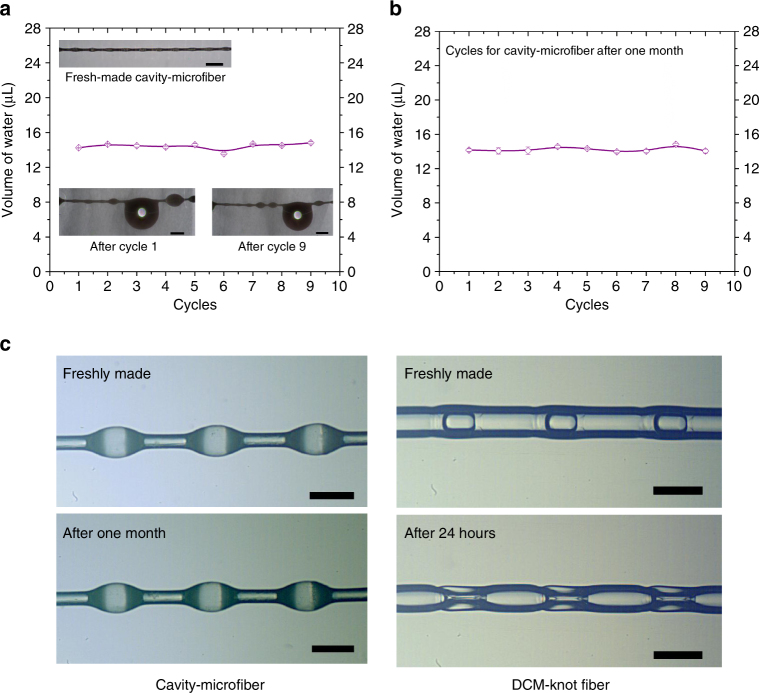
The durability of cavity-microfibers. **a** A plot of the maximum water volume collected by cavity-microfiber against the water-collecting cycles in each water-collecting cycle. Insets showing the cavity knots maintain their shapes after cycles of water collection. **b** A plot of the maximum water volume collected by cavity-microfiber stored for a month against the water-collecting cycles in each water-collecting cycle. **c** The cavity-microfiber maintains its morphology after 1 month, while the knots of the dichloromethane-droplet-templated fiber (DCM-knot fiber) deform greatly after 24 h. All error bars in (**a**) and (**b**) indicate the standard deviations over five independent measurements. Scale bars, 1 mm in (**a**) and 400 μm in (**c**)

Owing to its robustness, the cavity-microfiber can maintain the shape of cavity knots and collect water for cycles (Fig. 4a and see three separate water-collection processes in Supplementary Fig. 17). We examine the durability of cavity-microfiber for a month, during which the cavity shape, the water-collecting capability and mechanical strength are the same as those of fresh-made ones. The water-collection process of a 1-month-old cavity-microfiber (Supplementary Fig. 18) shows a similar linear increase of the collected water volume over time, and the maximum volume of water collected by a domain knot is also >10 μL, compared with Fig. 3c. Moreover, the cycling durability of water collection (Fig. 4b) and mechanical tensile strength (Supplementary Fig. 19) of the cavity-microfibers are almost not changed after 1 month, as demonstrated by the comparison in Fig. 4a, b and Supplentmary Fig. 7 and 19. In contrast, the shape of the knots formed by oil droplets shrinks greatly (Fig. 4c), which deteriorates its functionality. Therefore, the long-term durability of our cavity-microfibers enables the repeated water collection over time.

### Water collection of cavity-microfiber topological networks

**Fig. 5:**
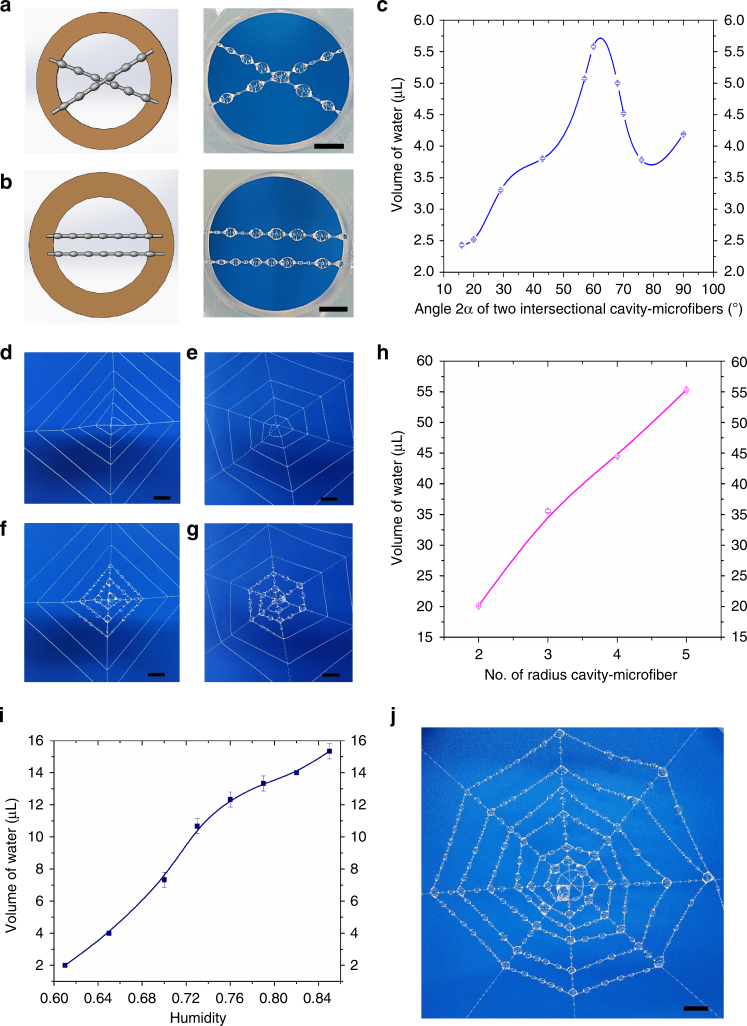
Water collection of cavity-microfiber topological networks. **a** The schematic diagram and water collection of intersectional structure of cavity-microfiber (fog flow: 0.408 mL min^−1^). **b** The schematic diagram and water collection of parallel structure of cavity-microfiber as contrast experiment (fog flow: 0.408 mL min^−1^). **c** A plot of the water volume collected by the two intersectional cavity-microfibers with the angle 2*α* against the angle (fog flow: 0.408 mL min^−1^). **d** Spider-web-like cavity-microfiber topological networks with two radius cavity-microfibers. **e** Spider-web-like cavity-microfiber topological networks with three radius cavity-microfibers. **f** Water collection of topological networks with two radius cavity-microfibers in (**d**) (fog flow: 0.408 mL min^−1^). **g** Water collection of topological networks with three radius cavity-microfibers in (**e**) (fog flow: 0.408 mL min^−1^). **h** A plot of the water volume collected by the topological networks against the number of radius cavity-microfiber (The cumulative length of cavity-microfiber: ~ 80 mm, collection time: 30 s; fog flow: 0.408 mL min^−1^). **i** The relationship between the humidity and the volume of water collected by cavity-microfiber-networks under airtight environment with no air convection. **j** Cavity-microfiber-networks with four radius cavity-microfibers to simulate the large-scale water collection. All error bars in (**c**), (**h**) and (**i**) indicate the standard deviations over five independent measurements. Scale bars, 3 mm in (**a**, **b**), 5 mm in (**d**–**g**) and **(j**)

To further increase the water capacity of assembled cavity-microfibers, we intersected two cavity-microfibers into an angle 2*α*. Comparing with two parallel cavity-microfibers, the intersectional configuration substantially increases the length of TCL (Supplementary Fig. 20a, b), and leads to a higher water capacity. Indeed, the water capacity of two intersectional cavity-microfibers is 8.25 μL (Fig. 5a), almost two times larger than that of two parallel cavity-microfibers, 4.57 μL (Fig. 5b), within 60 s. The water capacity is the highest at the intersectional angle *α* ≈ 30° (Fig. 5c). Moreover, if a water droplet is located on a knot near the intersection, it moves towards the intersection and coalesce with the droplet at the intersection. This is due to that the surface tension at the front-end of the droplet towards the intersection, ${\sigma _{\lg }}l{\rm{cos}}(\theta - \alpha )$ is always larger than that at the rare-end away from the intersection, ${\sigma _{\lg }}l\cos (\theta + \alpha )$ (Supplementary Fig. 21a, b and Supplementary Note 6)^15^. Thus, the water droplet transports directionally toward the intersection, where the water capacity is largest among any other locations on the cavity-microfiber.

To obtain more intersections for high-efficiency, we assembled the cavity-microfibers into topological networks, resembling the spider-web (Fig. 5d, e). The cavity-microfiber-networks were composed of radius threads and spiral threads. We crossed the radius cavity-microfibers for intersection configuration, and this intersection was the center of the network. Subsequently, we laid out more cavity-microfibers to form an auxiliary spiral, extending from the center to the outer edge of the network. We found that the number of the radius cavity-microfibers strongly affects the water-collecting capacity. Under the same cumulative length of cavity-microfibers for effective water collection (~ 150 mm) near the center of the network, the network composed by two radius cavity-microfibers has a water capacity of 53.297 μL (Fig. 5f), while that of three radius cavity-microfibers is about 68.957 μL (Fig. 5g), within the same time-period of 120 s. More radius cavity-microfibers lead to more intersections and thus stronger water-collecting capacity (Fig. 5h). Therefore, the efficiency of spider-web-like networks for water collecting can be significantly enhanced by tuning the topology of the network to have more nodes/intersections. In addition, the volume of water collected by cavity-microfiber-networks is larger for higher humidity. The volume of water collected by 86 cm-cumulative-length cavity-microfiber-networks with four radius cavity-microfibers in 300 s increases from 2 to 15.34 μL with the increase of humidity from 61 to 85% (Fig. 5i). Under a fog flow of 0.408 mL min^−1^ representing the fog in the early mornings of water-deficient areas, a cavity-microfiber-network of 77 cm-cumulative-length with four radius cavity-microfibers collect 0.36 mL water within 2 mins (Fig. 5j). Due to the cycling durability and the advantageous topological design, network of total area, ~ 0.079 m^2^, assembled by cavity-microfibers of equivalent length of ~ 28.49 m, can collect ~ 1 L water for 3 h (~ 25 cycles per hour) that can maintain vital signs of a person in a day.

## Discussion

In conclusion, we employ a simple microfluidic approach to fabricate spindle-knot microfibers with cavity knots for assembled topological networks and large-scale water collection. The spindle-knot structures such as the knot size and distance between knots are precisely controlled by the gas-phase pressure and flow-rate of jet phase. The cavity design as well as the polymer compositions enables the desirable surface roughness, mechanical strength and long-term durability. The maximum water volume collected on a single knot is almost 495 times than that of the knot on the cavity-microfiber we fabricated. On the basis of these results, the cavity-microfibers are assembled controllably into spider-web-like networks for large-scale water collection. To maximize the water-collecting capacity, the topology of the network should be designed to have as many nodes/intersections as possible. Our light-weighted yet tough, low-cost microfibers with high efficiency in directional water transportation creates opportunities for large-scale water collection. Moreover, the spider-silk-like microfibers with cavity-knots, as well as the assembled networks are potential candidates for tissue engineering, encapsulation and controlled release, and controlled liquid transport.

## Methods

### Fabrication of cavity-microfiber via microfluidic method

In a capillary-based microfluidic device, we employed ABC solution as the continuous phase, which was composed of PEG (4.6 wt %, Mn = 6000, Sigma-Aldrich), PVA (4.6 wt %, Mw = 13,000–23,000, 87–89% hydrolyzed, Sigma-Aldrich) and sodium alginate (2.7 wt %, Sigma-Aldrich). The nitrogen gas was used as the dispersed phase. High-precision syringe pump (LSP01-2A, LongerPump) and home-made gas pressure controller were used to inject ABC solution and nitrogen gas, respectively. The resultant jet wrapping the generated bubbles was collected in CaCl_2_ solution (5 wt %, CaCl_2_ anhydrous, powder, ≥97%, Sigma-Aldrich) to form solidified cavity-microfiber. A rotating collection roller collected solidified cavity-microfiber from the CaCl_2_ solution. The collected cavity-microfiber was dried at ambient temperature for more than 1 h to obtain the dehydrated cavity-microfiber with periodic spindle-knots and joints.

### Characterization of cavity-microfiber

The structures of the hydrated and dehydrated cavity-microfiber were observed with the inverted fluorescence microscope (Eclipse TS100, Nikon, Japan) equipped with a high-speed camera (Phantom, USA). The microscale morphology of the cavity-microfiber was further characterized by scanning electron microscopy (SEM; Hitachi S3400N VP, Japan). The surface roughness and nanostructures were characterized by atomic force microscope (Bruker NanoScope V). The tensile strength of microfibers was measured using Agilent T150 UTM Nano tensile test machine (Agilent Technologies, USA). The images analysis of the cavity-microfiber was performed using ImageJ software (http://rsb.info.nih.gov/ij/).

### Water-collecting behaviors

To investigate the water-collecting efficiency of dehydrated cavity-microfiber, a dehydrated cavity-microfiber was stuck on a home-made U-tape frame and placed in the upflow of water mist generated by an ultrasonic humidifier (Meiyueda HTJ-2061, Jiangmen HONETIAN Technology Co., LTD). The whole process of water collection was recorded by a high-speed camera (Phantom, USA).The average volume of water drops collected on a single spindle-knot at different collection times was measured by analyzing the snapshots of the recorded video. Both spindle-knots and water drops were considered as ellipsoids, and the water droplet volume ($V = \frac{4}{3}\pi abc$) was calculated based on the lengths of major axis (2*a*) and minor axis (2*b* = 2*c*) of the droplet.

To investigate the relationship between the humidity and water-collection ability of a single dehydrated cavity-microfiber and cavity-microfiber-networks, a sufficiently large airtight container was employed. The flow of water mist was used to adjust the air humidity in the container to desired values under the monitoring of a hygrometer (TH101B, GEMlead). A single dehydrated cavity-microfiber was stuck on a home-made U-tape frame in the airtight container for 10 mins; and the cavity-microfiber-networks were stuck on a home-made ring-shaped frame in the airtight container for 5 mins to collect water. We ensured no air convection was induced in the container during the process. The cavity-microfiber was then weighted to calculate the amount of water collected on a high-precision electronic scale (0.01 mg, MS105, METTLER TOLEDO) before and after the water collection. Water density, 1 g cm^−3^, was used in calculating the volume of water collected.

To investigate the water-collecting efficiency of two intersectional cavity-microfibers and cavity-microfiber-networks, we compared two dehydrated cavity-microfibers in intersectional and parallel configurations for their water-collecting efficiency. We placed the cavity-microfibers, as well as cavity-microfiber-networks, in the upflow of water mist generated by an ultrasonic humidifier (Meiyueda HTJ-2061, Jiangmen HONETIAN Technology Co., LTD). The collecting processes were recorded by a commercial camera and analyzed by ImageJ software (http://rsb.info.nih.gov/ij/).

### Data availability

The data that support the findings of this study are available from the corresponding author on request.

## Electronic supplementary material

Supplementary Information

Description of Additional Supplementary Files

Supplementary Movie 1

Supplementary Movie 2
