# Supplementary material for: Large-scale water collection of bioinspired cavity-microfibers
Source: Nat Commun. 2017 Oct 20;8:1080. doi: 10.1038/s41467-017-01157-4 (PMC5714965; doi:10.1038/s41467-017-01157-4)
Supplement: Supplementary file 1 — Supplementary Information [file 41467_2017_1157_MOESM1_ESM.pdf]

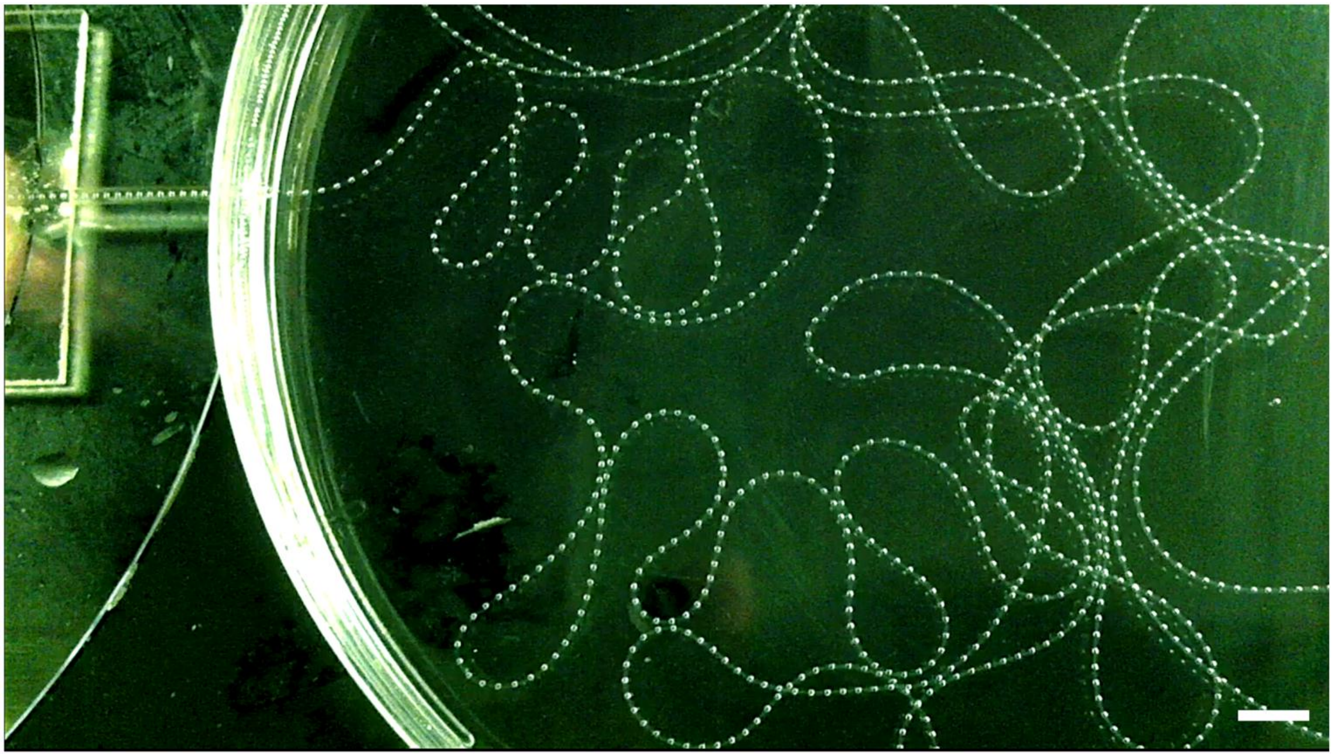

Supplementary Figure 1 | The optical image showing the fabrication of the cavity-microfiber. The ABC solution wrapping gas bubbles was extruded from the orifice into $CaCl_2$ solution and ABC solution can be crosslinked instantly into gelatinized alginate calcium fiber upon touching with the Ca^{2+} ions. Scale bar, 4 mm.

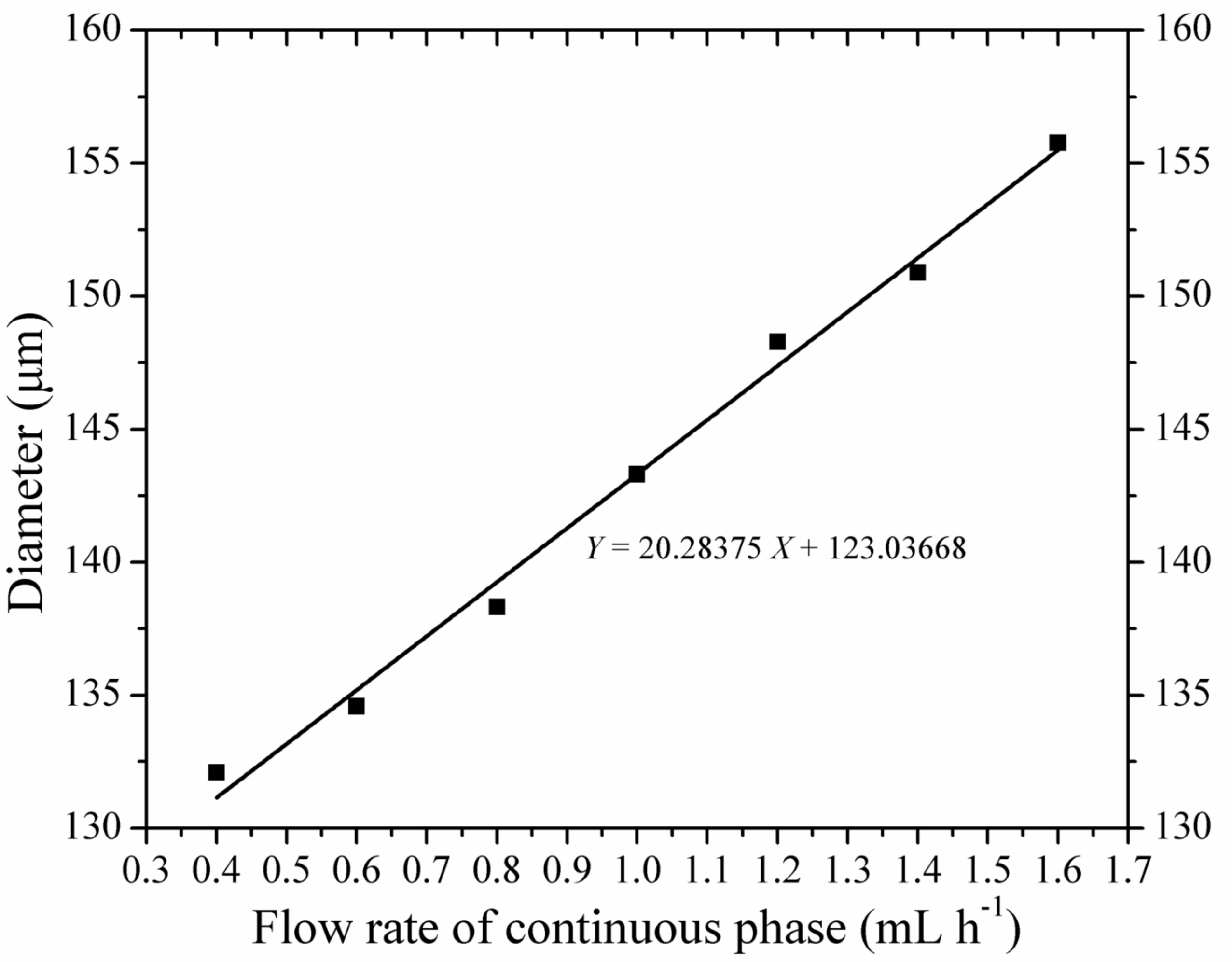

Supplementary Figure 2 | A plot of the diameter of hydrated cavity-microfiber against Q_{jet} **.** The diameter of hydrated cavity-microfiber was controlled by the flow rate of continuous phase, Q_{jet} .We varied Q_{jet} from 0.4 mL h^{-1} to 1.6 mL h^{-1}, and the diameter of hydrated cavity-microfiber increases from 132.093 μm to 155.768 μm linearly.

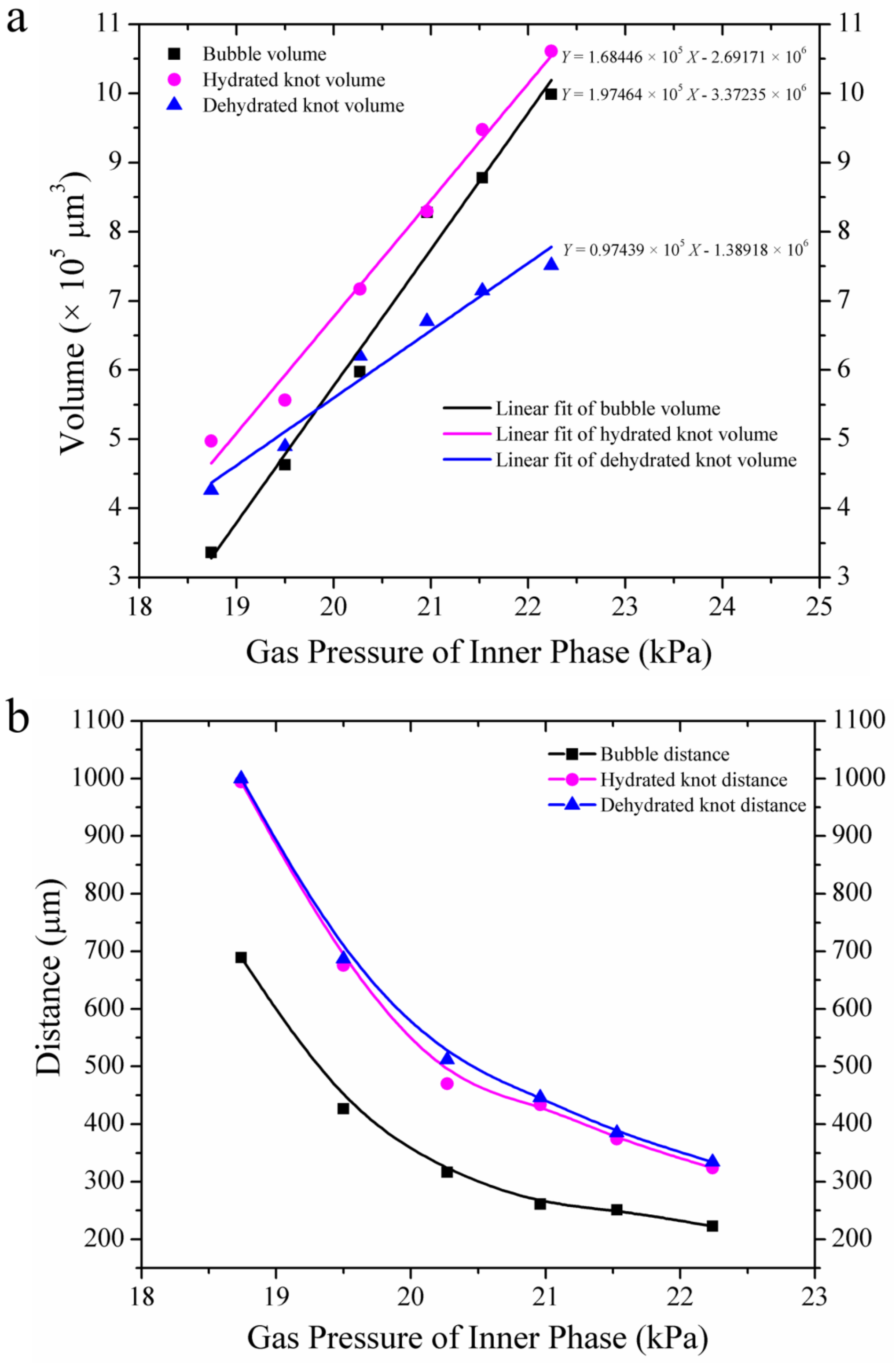

Supplementary Figure 3 | Effects of gas pressure on cavity-microfiber under Q_{jet} = 0.6 mL h^{-1}. (a) A plot of the volume of gas bubble, the knot volume of hydrated cavity-microfiber and the knot volume of dehydrated cavity-microfiber against the gas pressure of the dispersed phase, respectively. **(b)** A plot of the distances between two bubbles, two knots of hydrated cavity-microfiber and two knots of dehydrated cavity-microfiber against the gas pressure of the dispersed phase, respectively.

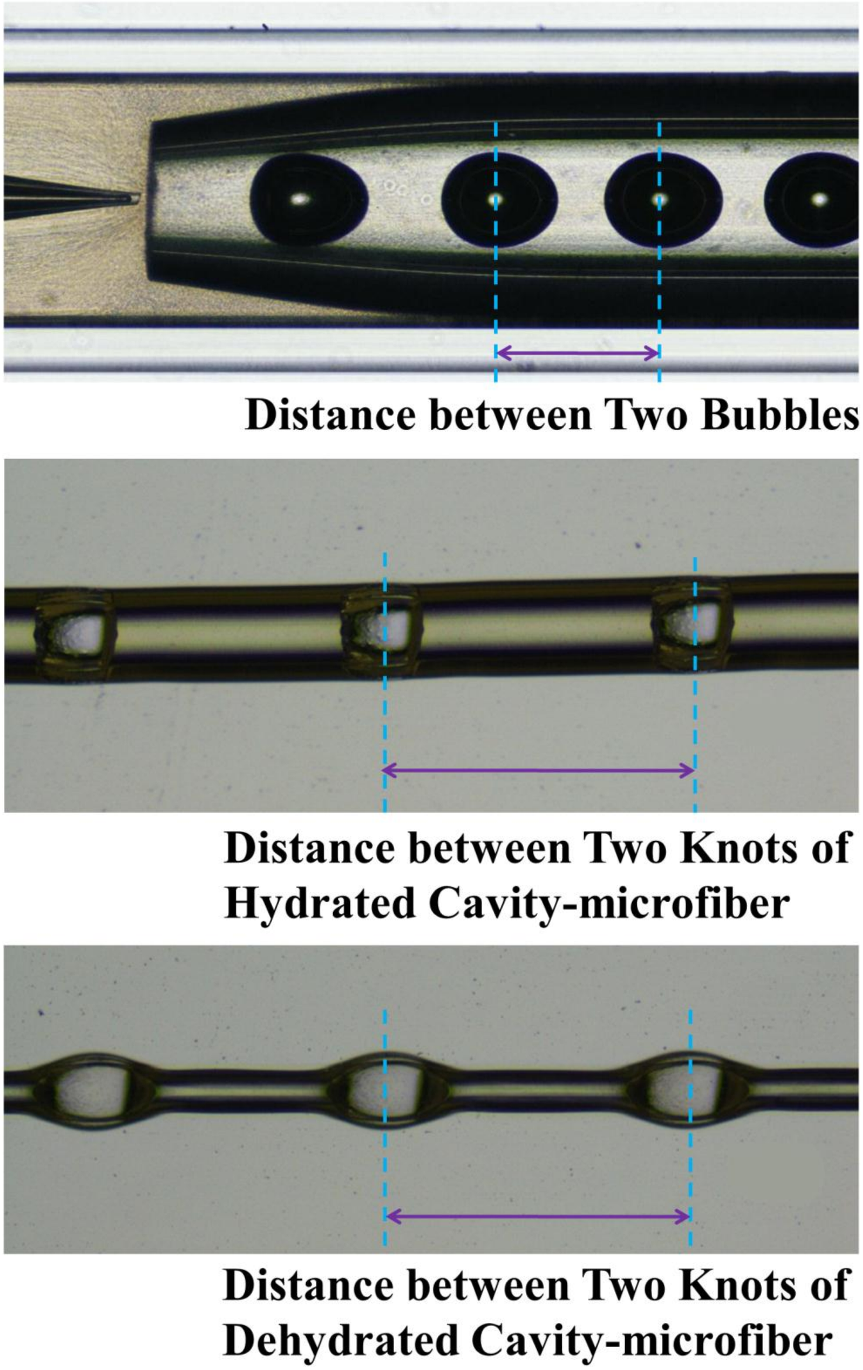

Supplementary Figure 4 | Diagram of three distance parameters. Optical images showing the distance between two bubbles, the distance between two knots of hydrated cavity-microfiber, and the distance between two knots of dehydrated cavity-microfiber.

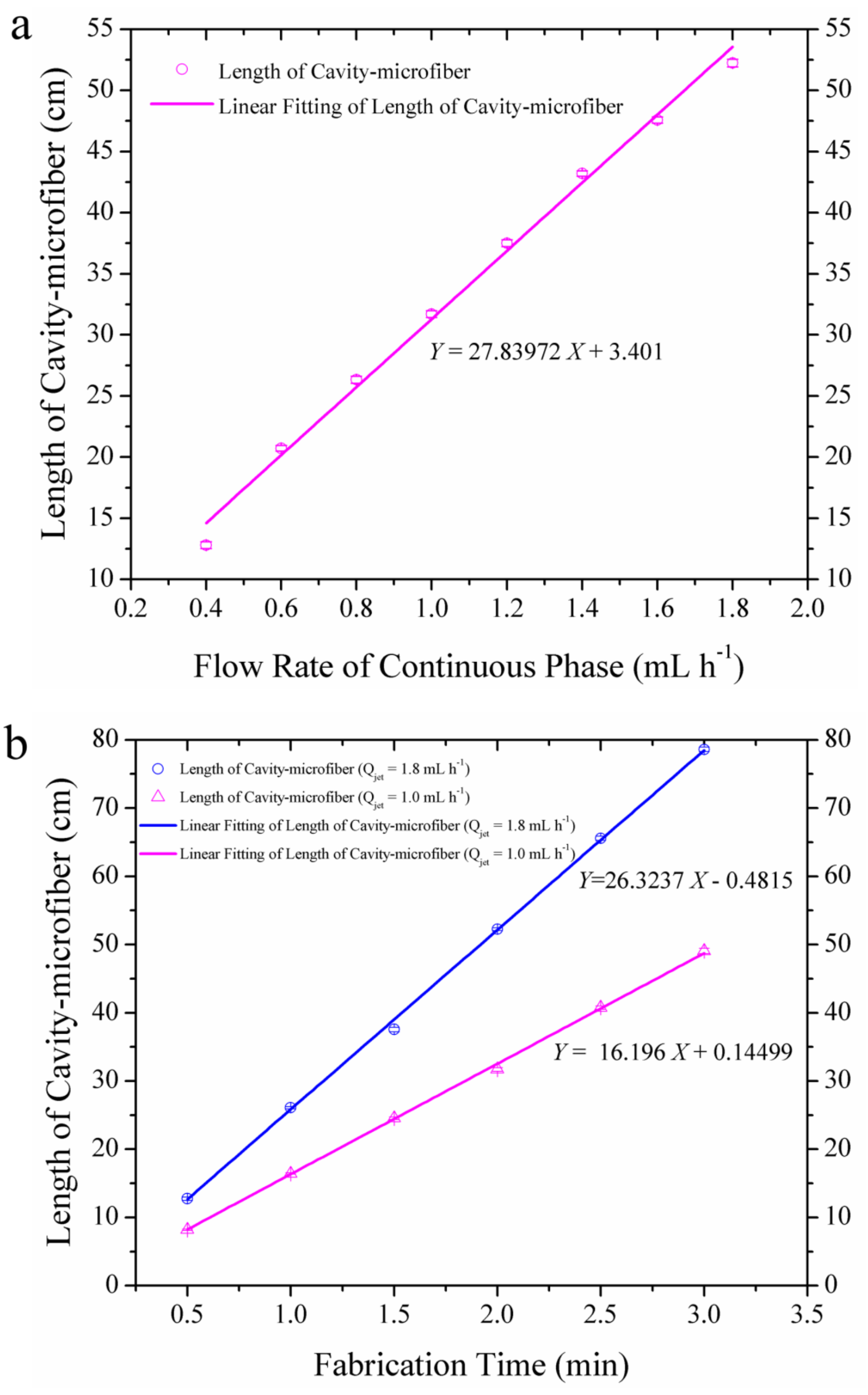

Supplementary Figure 5 | The production rate of cavity-microfiber. (a) A plot of the length of produced cavity-microfiber against the flow rate of continuous phase within 120s. **(b)** A plot of the length of produced cavity-microfiber against the fabrication time under the fixed Q_{jet} = 1.8 mL h^{-1} and Q_{jet} = 1 mL h^{-1}, respectively. All error bars in **(a)** and **(b)** indicate the standard deviations over five independent measurements.

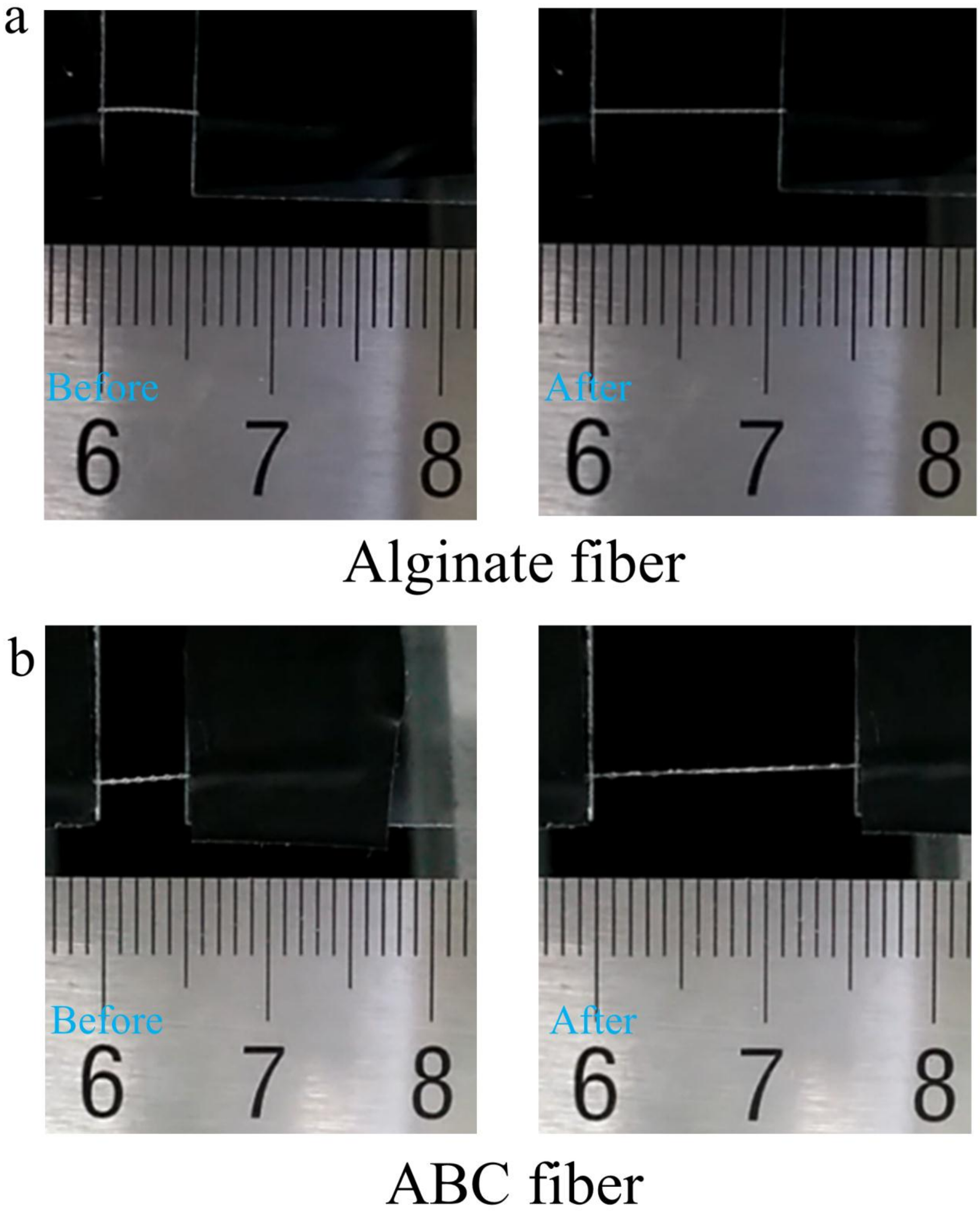

Supplementary Figure 6 | Mechanical strength of alginate fiber and ABC fiber. (a) The tensile test of alginate fiber (4% alginate solution). **(b)** The tensile test of cavity-microfiber (ABC solution). Both alginate fiber and cavity-microfiber have the same length of 5 mm. After tensile test, the alginate fiber has a cumulative length of ~10 mm, while that of cavity-microfiber is about 16 mm, within the same tensile conditions. (ABC solution means Alginate-based composite solution: 4.6 wt % PEG, 4.6 wt % PVA and 2.7 wt % sodium alginate).

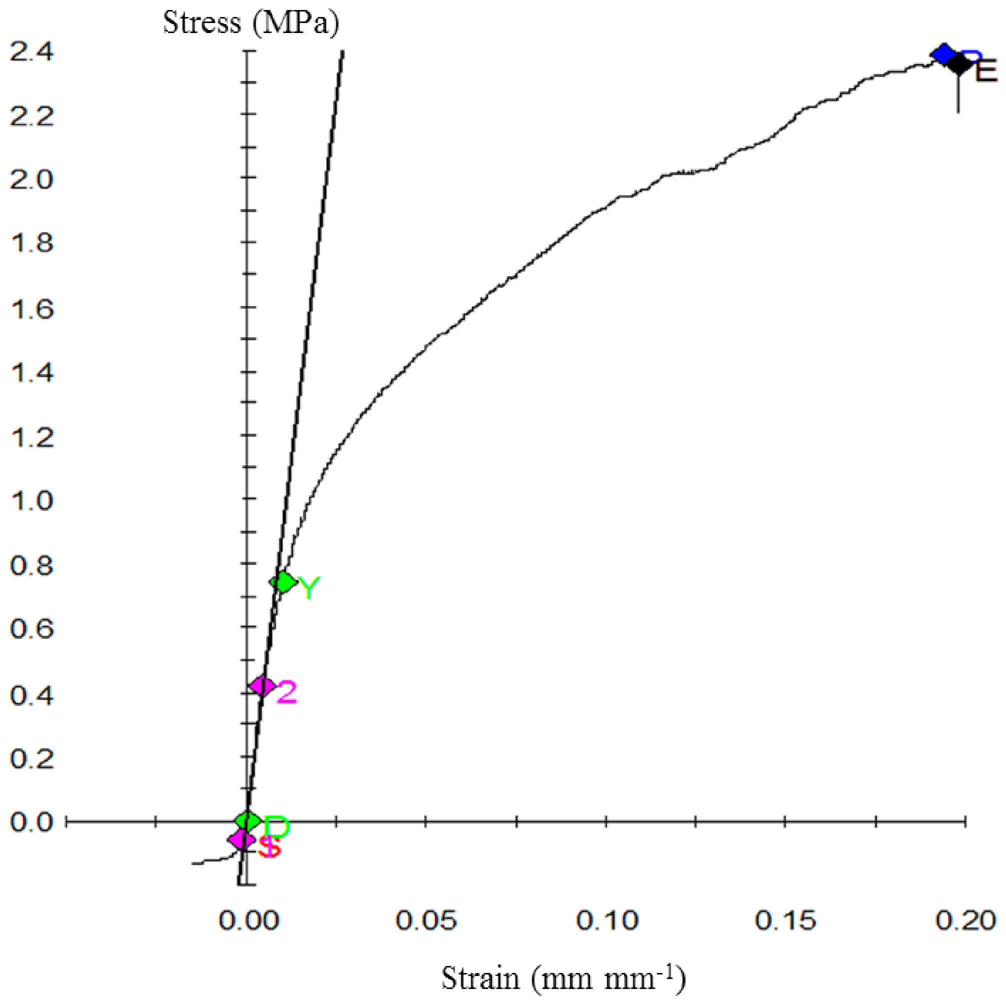

Specimen Diameter	Gage Length	Modulus	Offset Yield Stress	Offset Yield Strain	Max. Engineering Stress	Max. Engineering Strain
128 µm	20 mm	0.09 GPa	0.74 MPa	0.1 mm mm^{-1}	2.238 MPa	0.199 mm mm^{-1}

Supplementary Figure 7 | The tensile test of dehydrated cavity-microfiber. The maximum stress of resultant cavity-microfiber is 2.238 MPa.

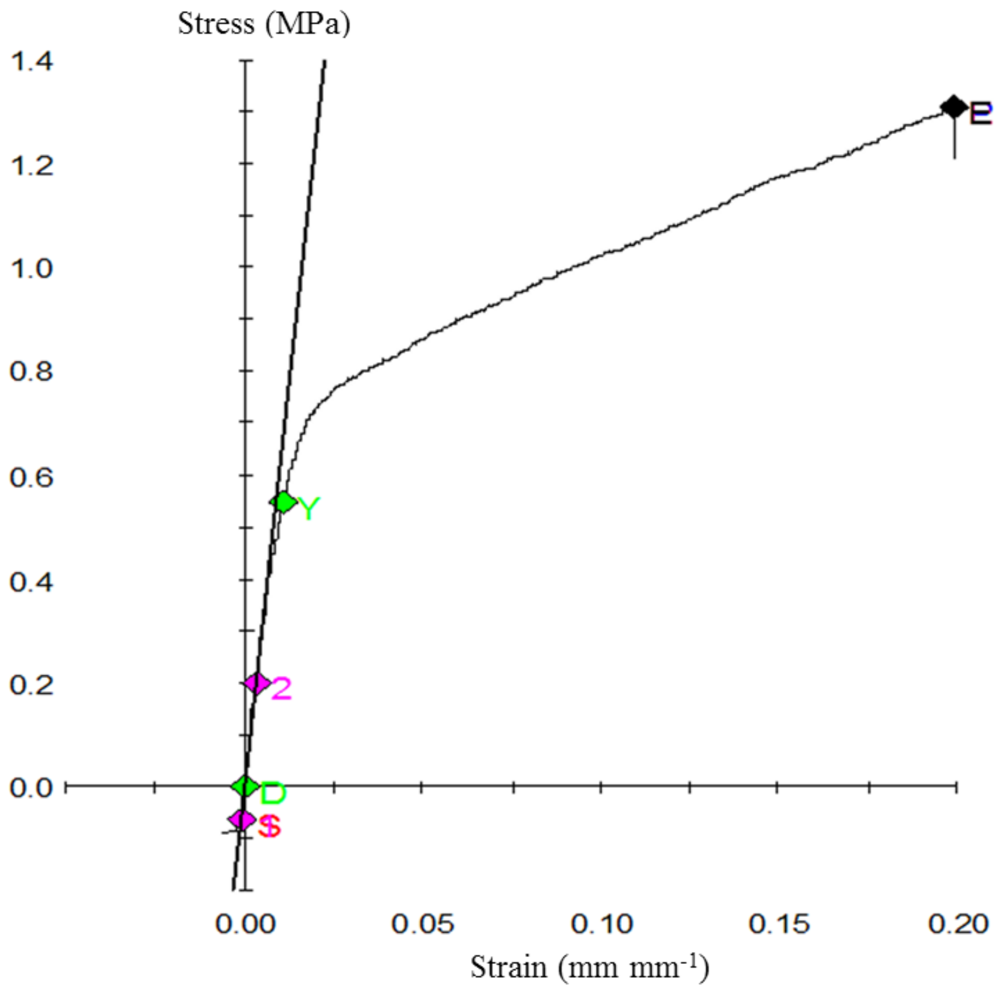

Specimen Diameter	Gage Length	Modulus	Offset Yield Stress	Offset Yield Strain	Max. Engineering Stress	Max. Engineering Strain
128 µm	20 mm	0.061 GPa	0.548 MPa	0.107 mm mm^{-1}	1.31 MPa	0.199 mm mm^{-1}

Supplementary Figure 8 | The tensile test of alginate fiber. The maximum stress of alginate fiber is 1.31 MPa, less than that of cavity-microfiber, 2.238 MPa.

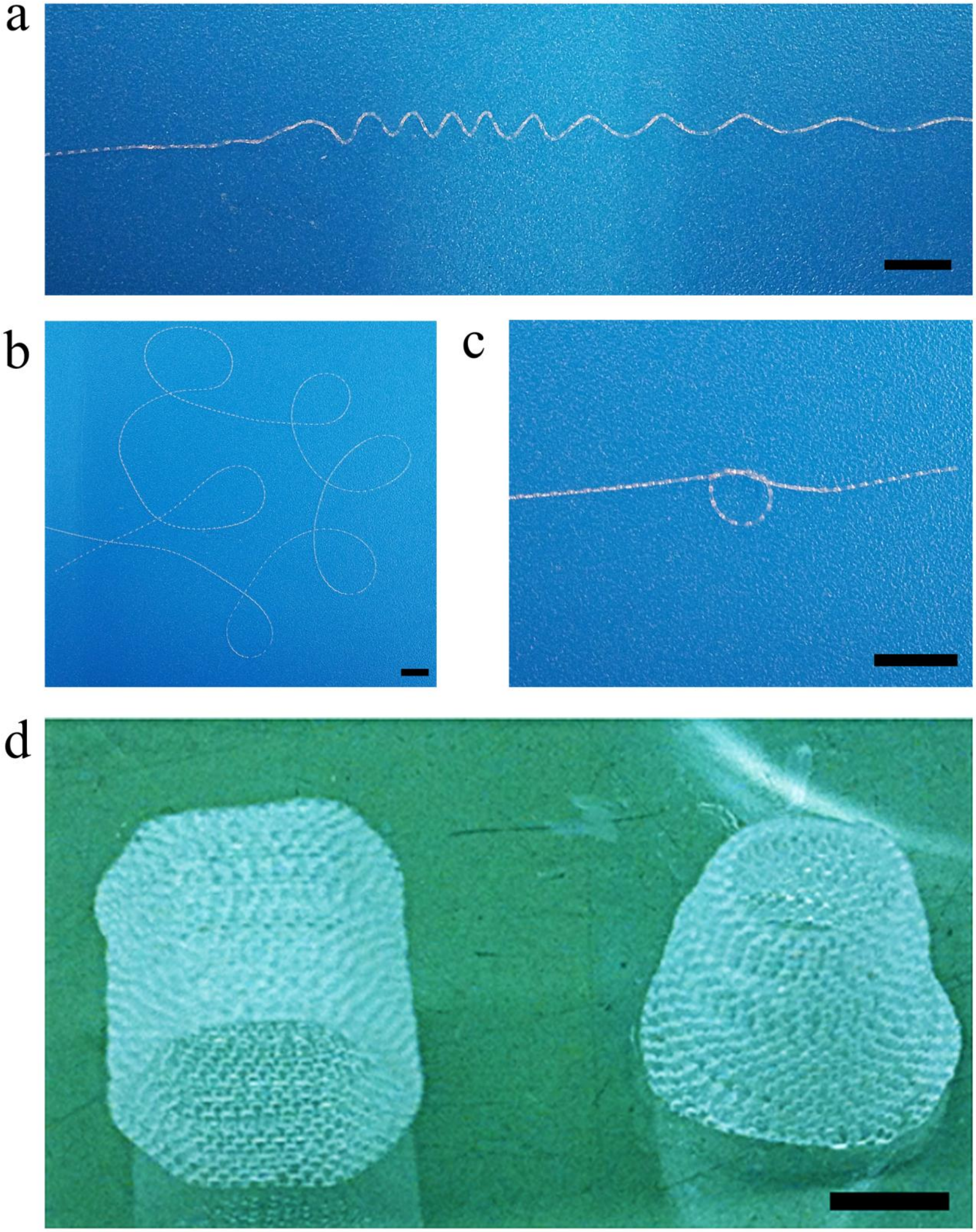

Supplementary Figure 9 | The bending property of cavity-microfiber. The cavity-microfiber can be easily **(a)** spiralled, **(b)** folded, **(c)** tied and **(d)** assembled into 3D scaffolds. Scale bars, 5 mm.

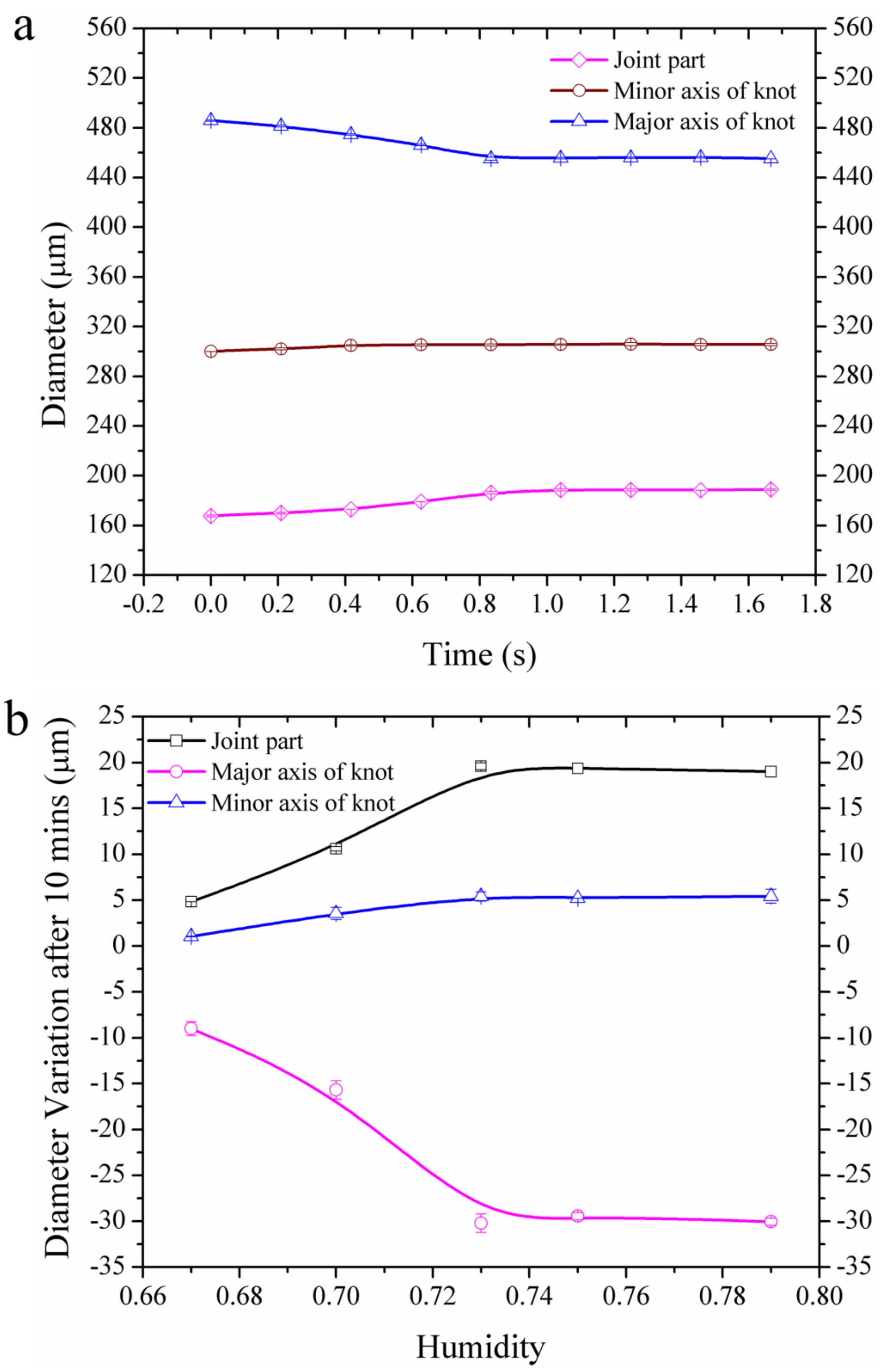

Supplementary Figure 10 | The swell of cavity-microfiber. (a) Plot of evolution of the diameter of joint part, the diameter of minor axis of knot and the diameter of major axis of knot due to the swell of cavity-microfiber soaked into water. **(b)** Plot of variations of diameter for the joint part, minor axis of knot and major axis of knot, respectively, with the humidity, within 10 mins (Diameter after swell – Original diameter). All error bars in **(a)** and **(b)** indicate the standard deviations over five independent measurements.

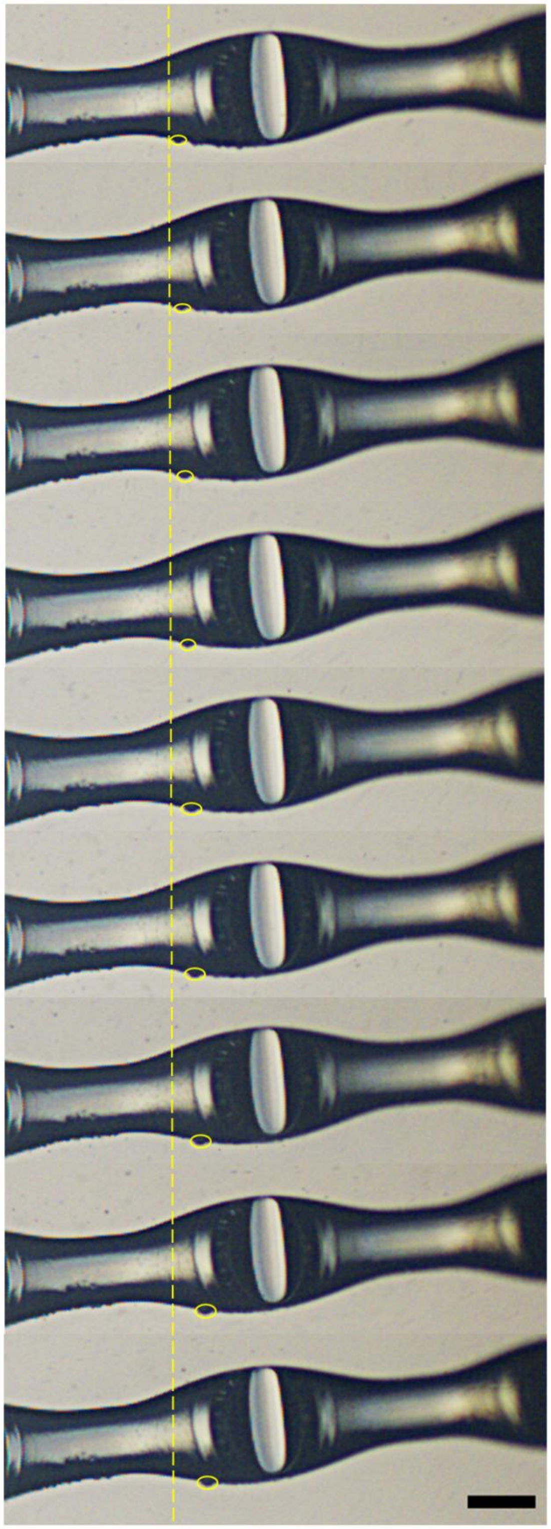

Supplementary Figure 11 | Tiny water droplets move from the joint to the knot. Due to the surface energy gradient and Laplace pressure, the tiny water droplets can move directionally from the joint part to the knot part to form larger water droplet (The interval: 71.42855 ms). Scale bar, 200 μm.

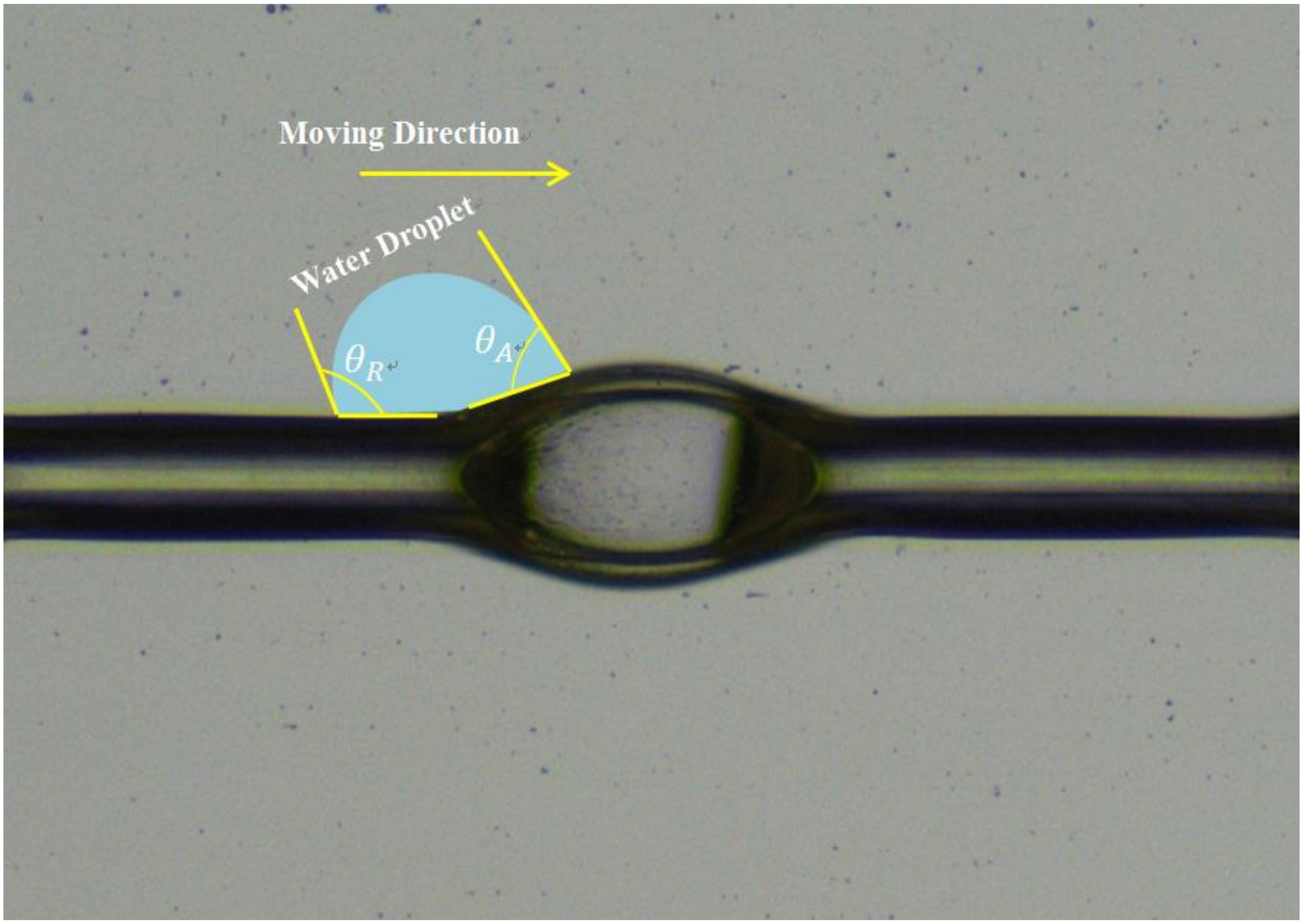

Supplementary Figure 12 | Analysis of the driving force arising from surface energy gradient. The force points towards the direction from the joint with lower surface energy to the spindle knot with higher surface energy.

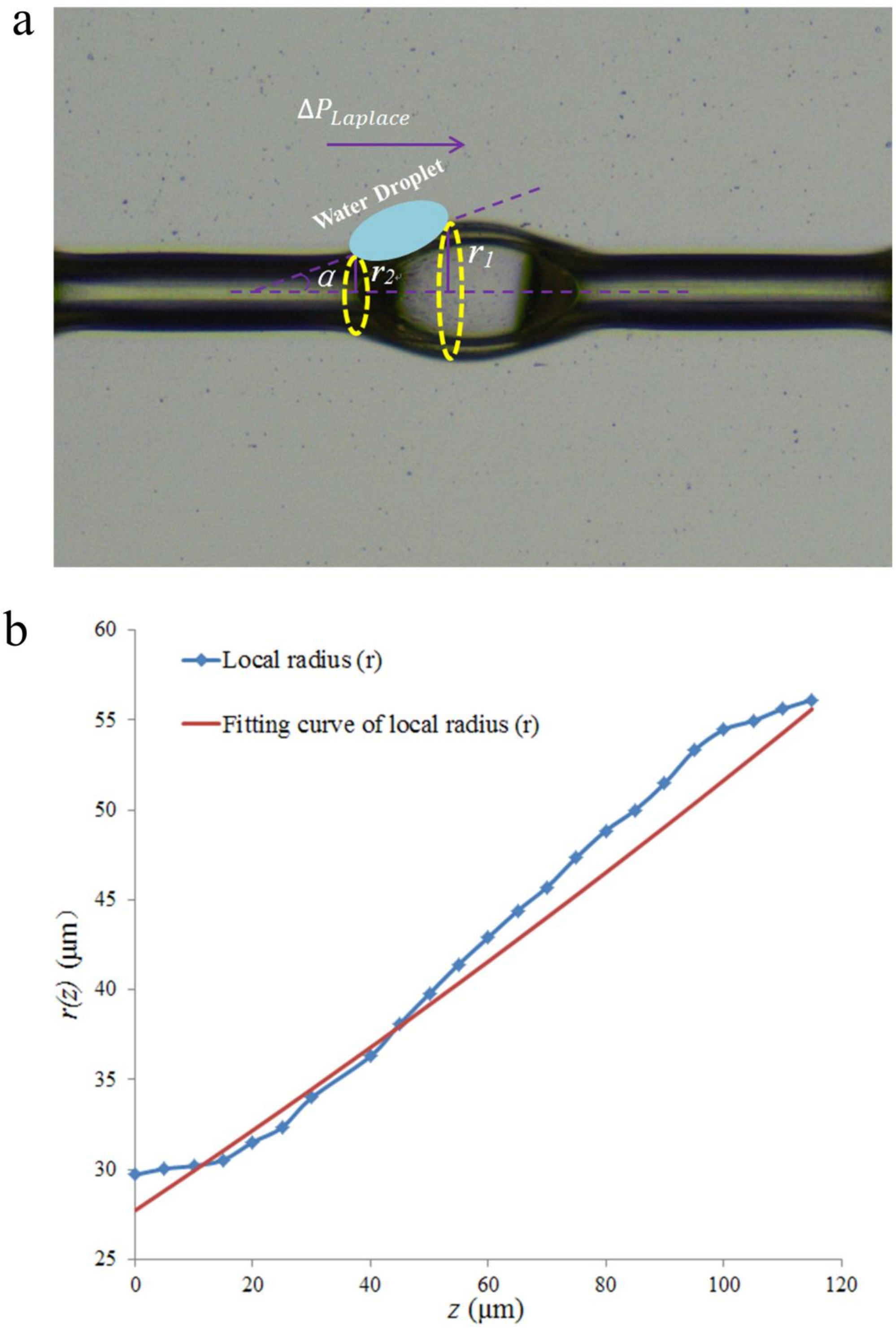

Supplementary Figure 13 | Analysis of the driving force arising from the difference of Laplace pressure. **(a)** Schematic diagram of the driving force arising from the difference of Laplace pressure. **(b)** Relationship between the position variable (z) along the axial direction of dehydrated cavity-microfiber and the local radius (r) of dehydrated cavity-microfiber based on the experimental data, and its fitting curve.

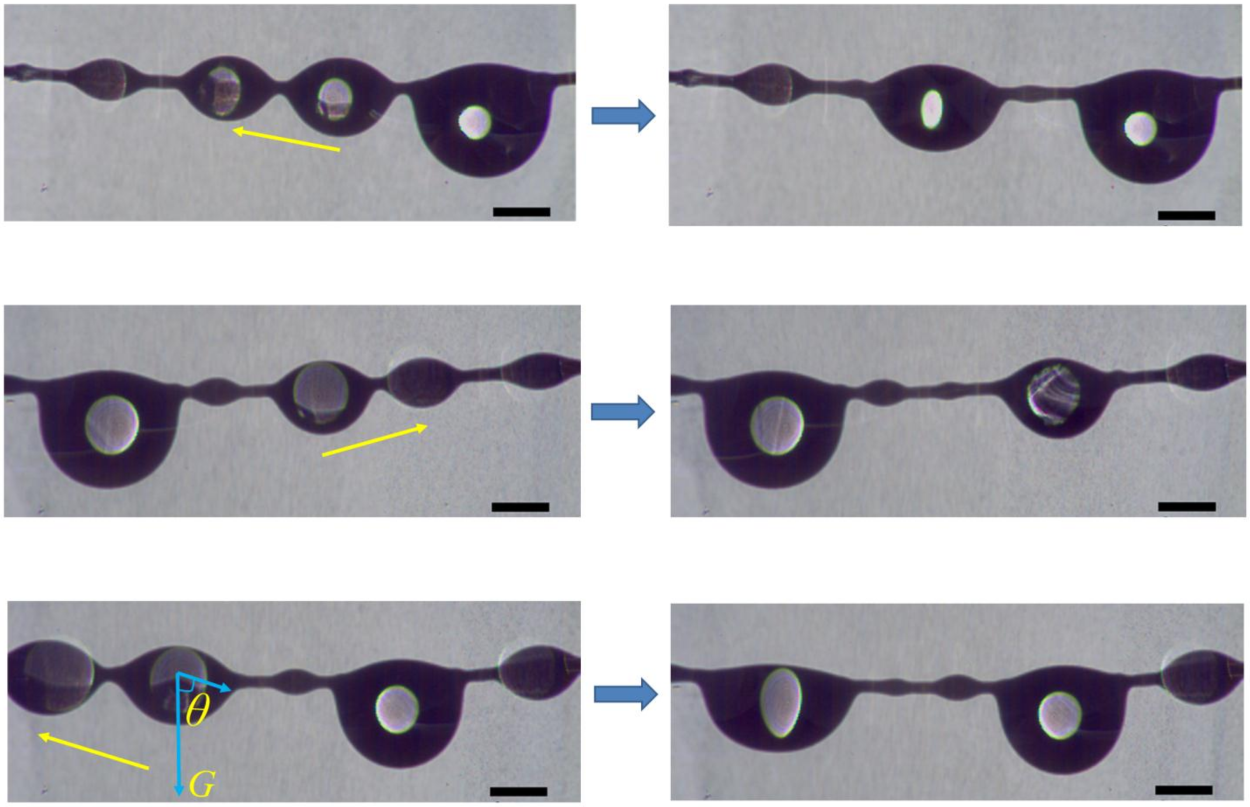

Supplementary Figure 14 | The water droplets move up to the domain knots. Three examples show the water droplets moving up to domain knots. G is the gravity force of water droplet and θ is the angle between the gravity and longitudinal direction of cavity-microfiber. Scale bars, 1 mm.

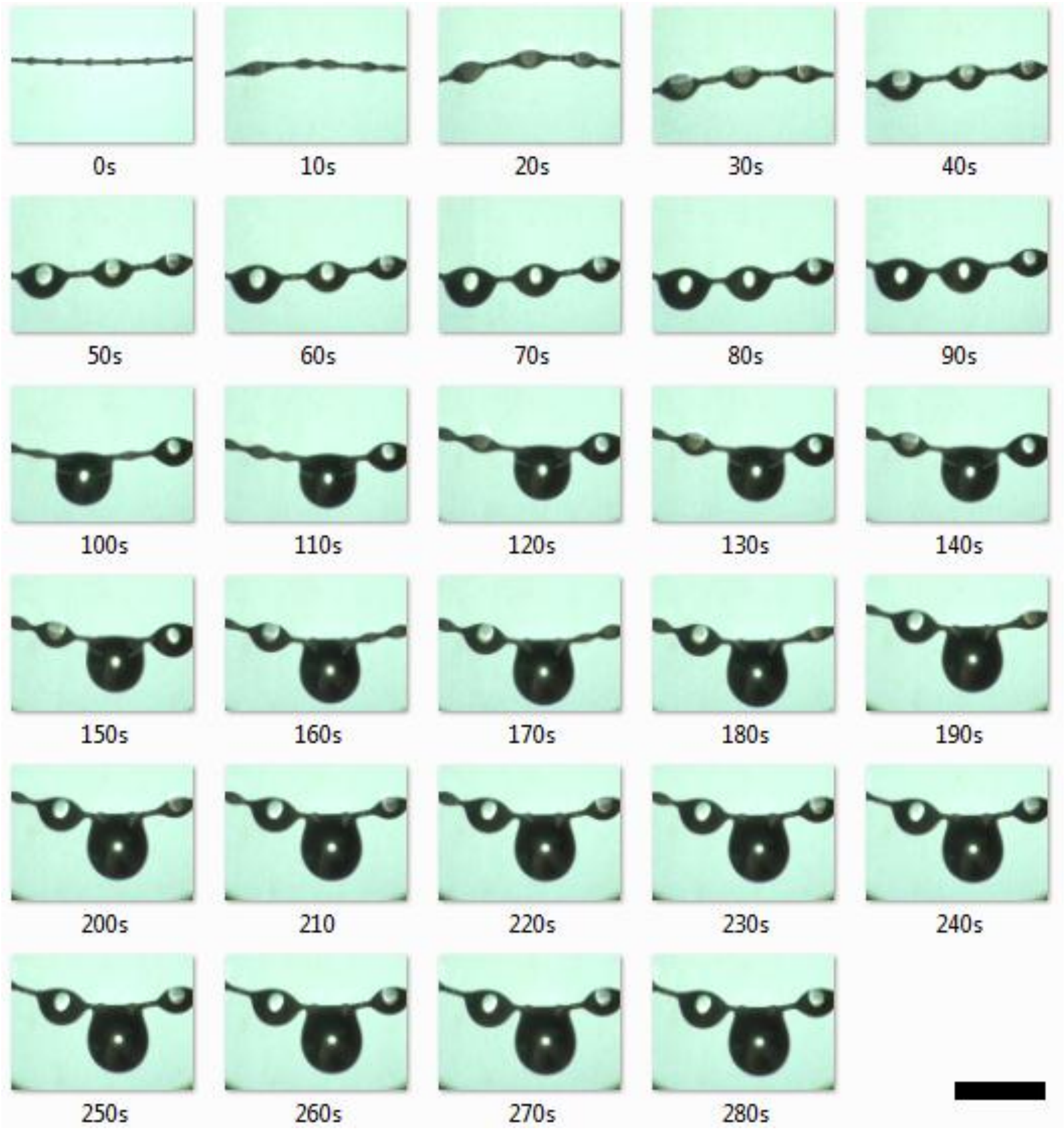

Supplementary Figure 15 | Time-sequence series of optical images showing the water-collection process. The water collected on a domain knot is shown over the time. The water droplets moved towards the domain knot, thus a larger droplet was collected. Scale bar, 4 mm.

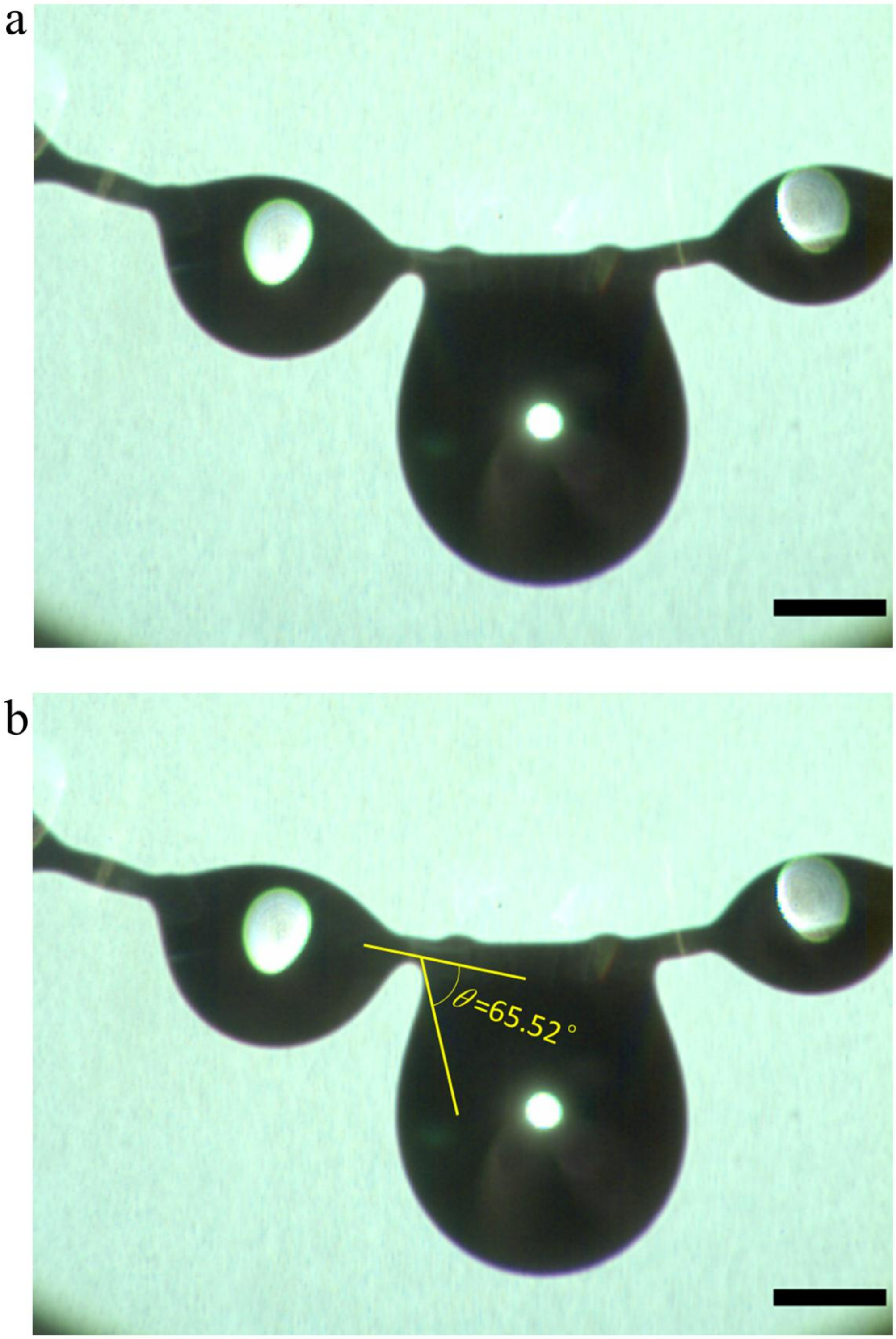

Supplementary Figure 16 | The maximum volume of water droplet collected by the domain knot. **(a)** Optical image showing the maximum volume of water droplet collected by the domain knot. **(b)** The contact angle of the water droplet with maximum volume on the cavity-microfiber is ~65.52°. Scale bars, 1 mm.

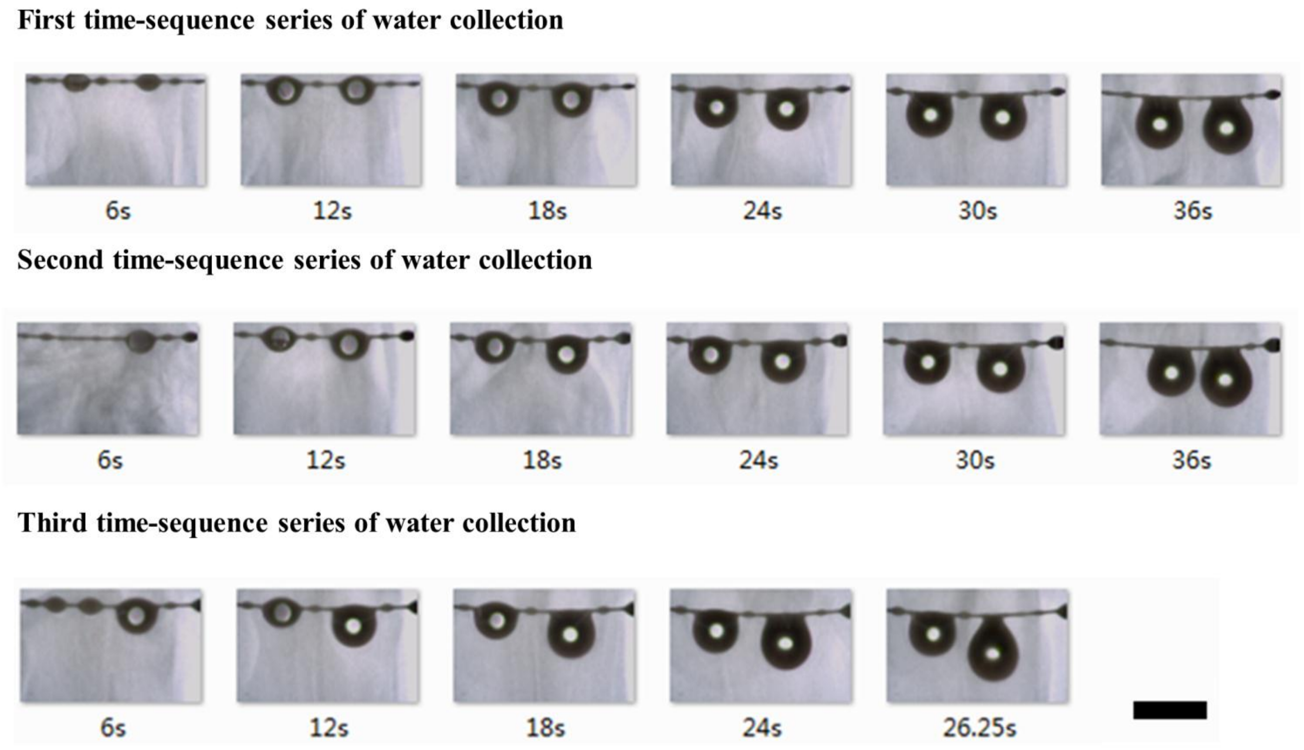

Supplementary Figure 17 | Time-sequence series of optical images showing three separate water collection processes. Three water collection processes on a domain knot are shown over time continuously. The fog flow used is fixed at 1.2744 mL min^{-1}. Scale bar, 4 mm.

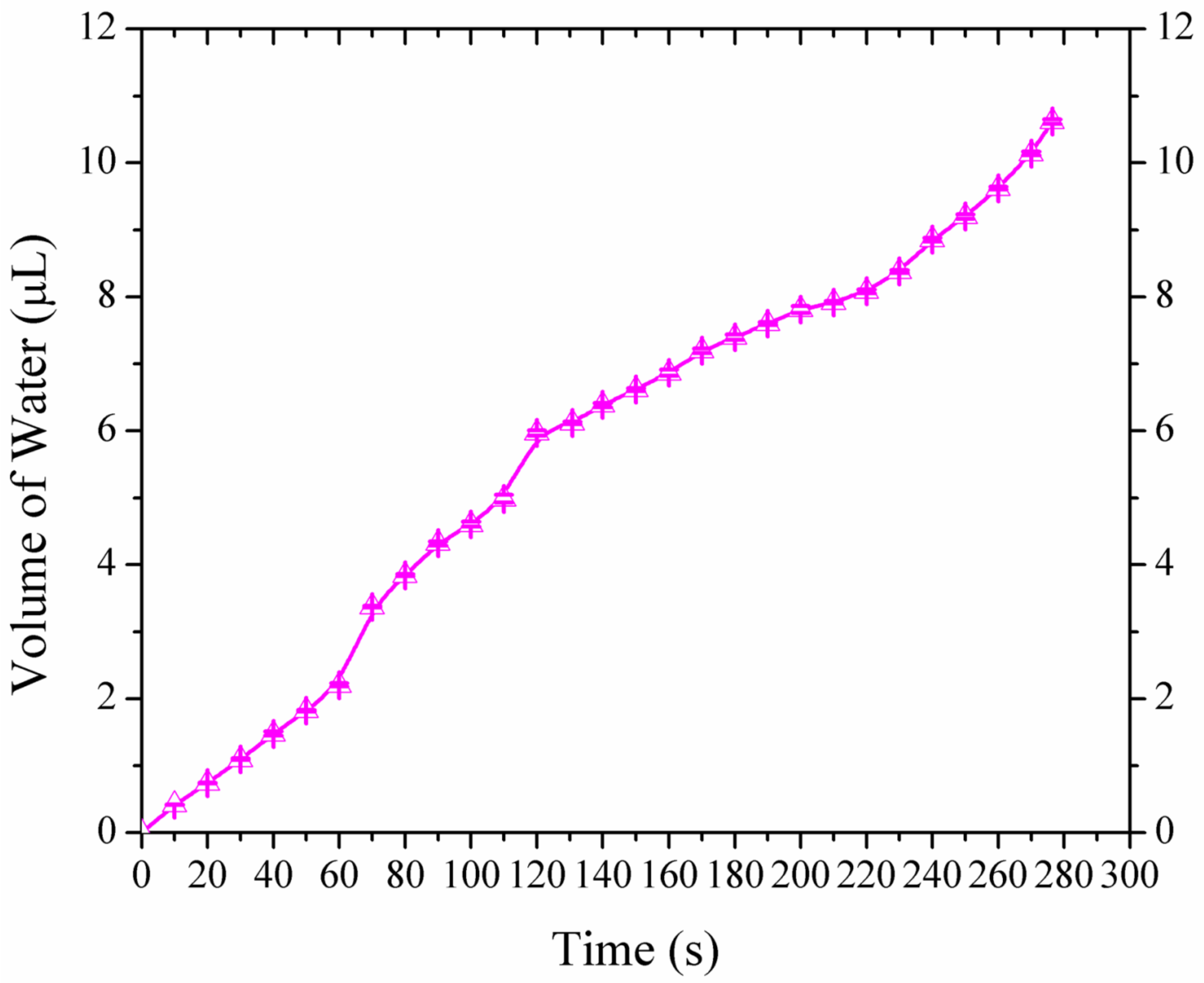

Supplementary Figure 18 | Water collection of dehydrated cavity-microfiber stored for a month. A plot of the volume of water collected against time by dehydrated cavity-microfiber stored for a month, showing a similar linear increase of the collected water volume over time, and the maximum volume of water collected by a domain knot, compared with Fig.3c. All error bars indicate the standard deviations over five independent measurements.

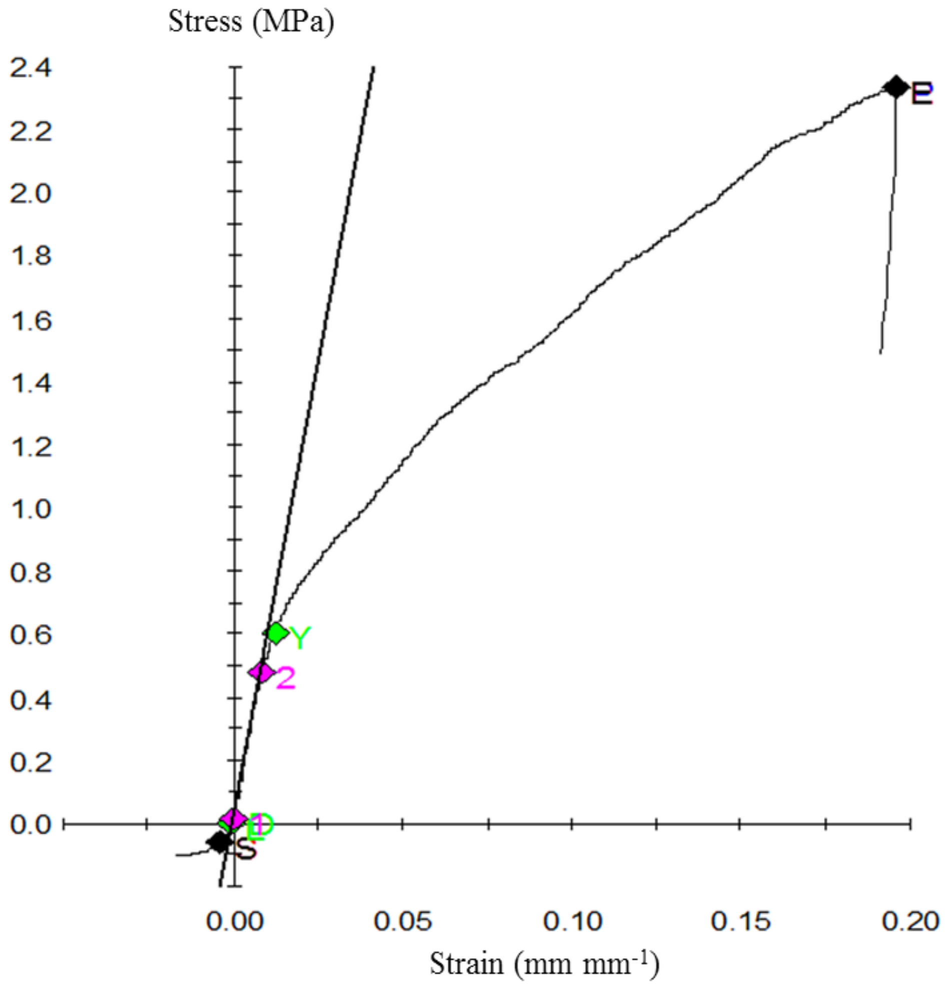

Specimen Diameter	Gage Length	Modulus	Offset Yield Stress	Offset Yield Strain	Max. Engineering Stress	Max. Engineering Strain
128 µm	20 mm	0.057 GPa	0.602 MPa	0.123 mm mm^{-1}	2.233 MPa	0.191 mm mm^{-1}

Supplementary Figure 19 | The tensile test of dehydrated cavity-microfiber stored for a month. The maximum stress of dehydrated cavity-microfiber stored for a month is 2.233 MPa, close to that of cavity-microfiber, 2.238 MPa in Supplementary Figure 7 and showing the durability of mechanical property of cavity-microfiber.

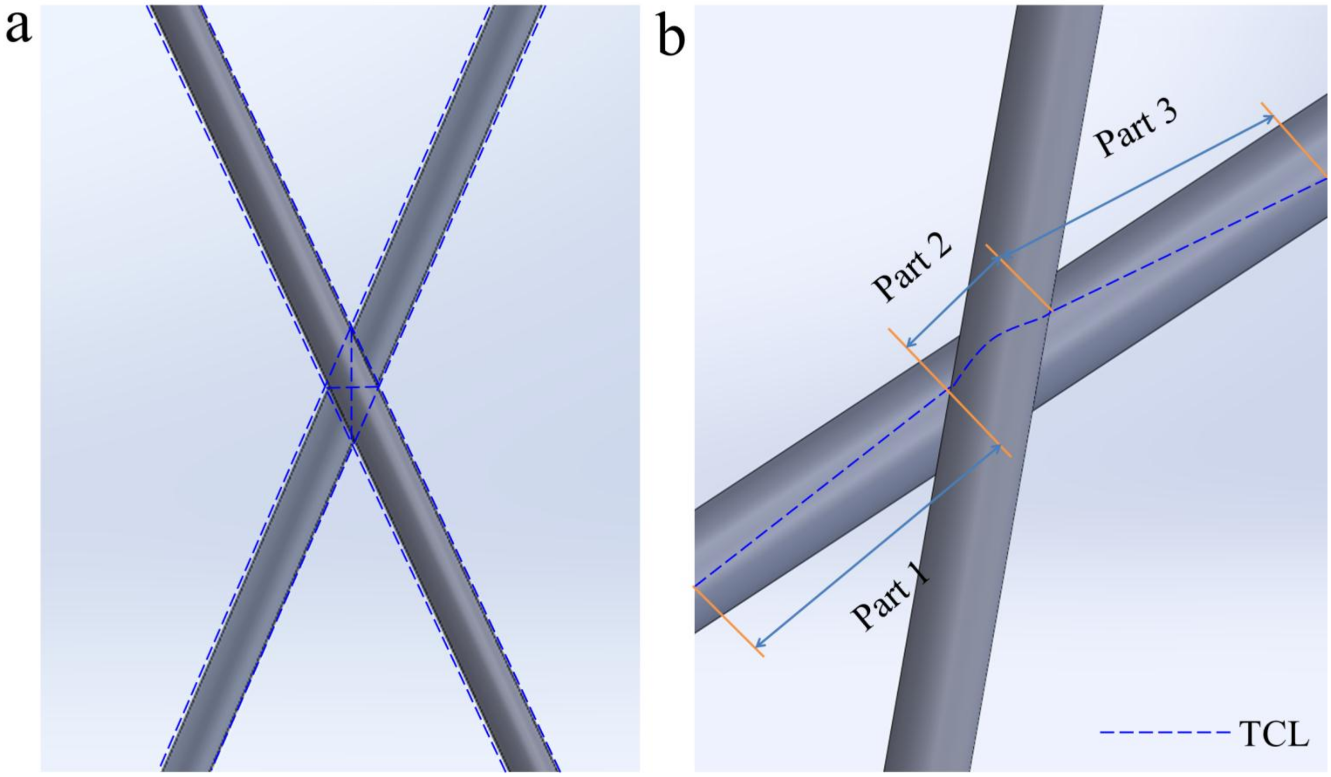

Supplementary Figure 20 | The schematic diagram of TCL of the intersectional cavity-microfibers. (a) The top view of TCL of the intersectional cavity-microfibers. **(b)** The side view of one TCL of the intersectional cavity-microfibers. The TCL length increases due to length increases of Part 1 and Part 3 (the slant contact lines), and Part 2 (the raised curve contact line due to the intersectional structure of cavity-microfibers).

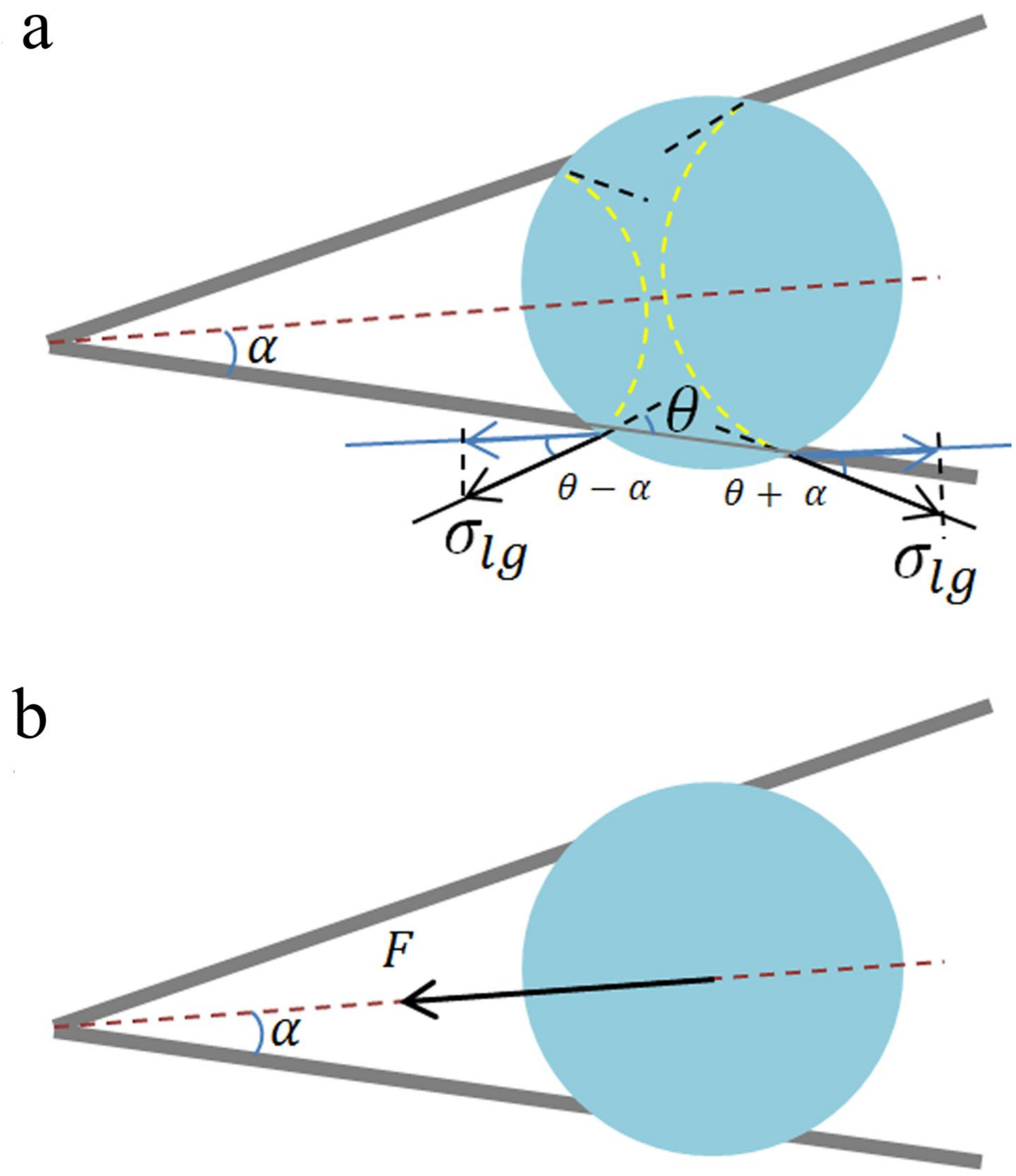

Supplementary Figure 21 | Driving force of water droplet on intersectional cavity-microfibers. (a) Analysis of the driving force of water droplet on the intersectional structure of cavity-microfiber only arising from the difference of liquid-gas interfacial tensions. **(b)** The direction of the driving force of water droplet on the intersectional structure of cavity-microfiber.

Supplementary Table 1-Comparison of production rate between our method and other methods

Methods / Production Rate / Reference	Production Rate in the Literature	Production Rate with Our Method
Ref.1	10-200 mm min^{-1}	64-at least 263 mm min^{-1}
Ref.2	0.6-180 mm min^{-1}	64-at least 263 mm min^{-1}

Supplementary Table 2-Comparison of water collection efficiency between cavity-microfibers and other fibers

Fiber Name / Water Volume / Time	Fibers in the Literature	Cavity-microfibers
170s (ref.3)	≈5.49 μL	≈9.08 μL
173s (ref.4)	≈6.76 μL	≈9.15 μL
210s (ref.5)	≈4.52 μL	≈9.72 μL
7.2s (ref.6)	≈0.00083 μL	≈0.043 μL
19s (ref.7)	≈0.365 μL	≈0.384 μL

Supplementary Table 3-Comparison of surface roughness between cavity-microfibers and other fibers

Fiber Name / Roughness / Reference	Fiber in the Literature	Cavity-microfiber
Ref.8	0.73 nm - 0.92 nm	133 nm – 204 nm

Note that the above as-compared data is not obtained under strictly identical conditions due to asynchronous experiments by different groups.

Supplementary Note 1-Analysis of the driving force arising from surface energy gradient.

The force arising from surface energy gradients may be one of the main driving forces. Due to no change about chemical composition of dehydrated cavity-microfiber, surface energy gradients can arise from surface roughness difference of dehydrated cavity-microfiber which can push water droplets to move towards higher surface energy, namely, more wettable region. According to Wenzel's law[3-7]:

$$\cos\theta_w = r\cos\theta \tag{1}$$

Where θ_w is the apparent contact angles, θ is the intrinsic contact angle, and r is the roughness factor defined as the ratio of the actual surface area to the geometric projected area of a rough surface. As for dehydrated cavity-microfiber, the joint part has smaller and more axial-parallel nanostructures so that there is larger water contact angle on the joint part than that of spindle knot of dehydrated cavity-microfiber (higher apparent surface energy than joint) [5].The driving force from surface energy gradient due to the difference of surface roughness of dehydrated cavity-microfiber (Supplementary Fig.12) can be expressed as follows[3, 5, 9, 10]:

$$\Delta F_{seg} = \int_{L_j}^{L_k} \gamma(l)(\cos\theta_A - \cos\theta_R)dl \tag{2}$$

where θ_A is the advancing angle of water droplet on dehydrated cavity-microfiber; θ_R is the receding angle of water droplet on dehydrated cavity-microfiber (Note: θ_A is less than θ_R based on Wenzel's law.); $\gamma(l)$ is the surface tension of water; l is the integrating variable along the length from the region close to joint part (L_j) to the region near the spindle knot (L_k). The force ΔF_{seg} points towards the direction from the joint with lower surface energy to the spindle knot with higher surface energy. Therefore, the force ΔF_{seg} can also push water droplet to move from joint to spindle knot.

Supplementary Note 2-Analysis of the driving force arising from the difference of Laplace pressure.

Water collection of dehydrated cavity-microfiber is highly dependent on the directional transport of the water droplets, resulting in larger and larger water droplet gathered on the spindle knot of dehydrated cavity-microfiber. One of the main driving forces is probably from the difference of Laplace pressure [5, 11, 12] caused by the curvature gradient of dehydrated cavity-microfiber (Supplementary Fig.13a). The difference of Laplace pressure can be expressed as:

$$\Delta P_{Laplace} = -\int_{z_1}^{z_2} \frac{2\gamma}{[r(z)+R_0]^2} \sin\alpha dz \tag{3}$$

where z is position variable (integrating variable) along the axial direction of dehydrated cavity-microfiber, starting from the end of joint part, z_1 is the position of the small local radius (r_1) of spindle knot, z_2 is the position of the large local radius (r_2) of spindle knot, $r(z)$ is local radius of dehydrated cavity-microfiber, R_0 is the radius of the water droplet, γ is the surface tension of water and α is the half apex-angle of the spindle knot of dehydrated cavity-microfiber. As r_1 is smaller than r_2, the Laplace pressure on the high curvature site (r_1, close to the joint of dehydrated cavity-microfiber) is larger than that on the low curvature site (r_2, close to the spindle knot of dehydrated cavity-microfiber). Namely, the difference of Laplace pressure points towards the center of spindle knot on the dehydrated cavity-microfiber. Therefore, the Laplace pressure gradient within the water droplet can act as a driving force to make the water droplet directionally transport from the joint part to the spindle knot of dehydrated cavity-microfiber so that the water droplet hanging on the spindle knot of dehydrated cavity-microfiber is becoming larger.

As for dehydrated cavity-microfiber (DCM), we can get the approximate relation between z and $r(z)$ from experimental data and calculate the $\Delta P_{Laplace}$ of DCM (Supplementary Fig.13b):

$$r(z) \approx A + Be^{kz} \tag{4}$$

$$\Delta P_{LaplaceforDCM} \approx -\int_{z_1}^{z_2} \frac{2\gamma}{[A+Be^{kz}+R_0]^2} \sin\alpha dz$$

$$= \frac{2\gamma \sin\alpha[-z_1+z_2-\frac{A+R_0}{k(A+Be^{z_1k}+R_0)}+\frac{A+R_0}{k(A+Be^{z_2k}+R_0)}+\frac{\ln(A+Be^{z_1k}+R_0)}{k}-\frac{\ln(A+Be^{z_2k}+R_0)}{k}]}{(A+R_0)^2}$$

where A, B and k are constant.

Supplementary Note 3-The gravity effect on the domain knot

Domain knot is determined by the difference in the surface energy and thus the Laplace pressure gradient. The domain knot is not determined by gravity. From Supplementary Fig. 14 and Supplementary Movie 2, water droplets can move up to the upper domain knot. The gravitational component along the cavity-microfiber ($F_{\mathrm{af}} = G\cos\theta$) is less than or equal to the driving force ($F_{\mathrm{d}} = \Delta F_{\mathrm{seg}} + \Delta P_{Laplace}$), making the droplet moving towards the domain knot.

If water droplet is sufficiently large, the cavity-microfiber can be bowed sharply and the angle between the gravity and longitudinal direction of cavity-microfiber is becoming smaller, resulting in a larger component of gravity along longitudinal direction of the cavity-microfiber ($F_{\mathrm{af}} = G\cos\theta$). As it overcomes the driving force ($F_{\mathrm{d}} = \Delta F_{\mathrm{seg}} + \Delta P_{Laplace}$), the water droplet moves to any knots at a lower position. Here G denotes the gravity force of water droplet, θ is the angle between the gravity and longitudinal direction of cavity-microfiber, ΔF_{seg} is the driving force from surface energy gradient due to the difference in surface roughness of cavity-microfiber (Supplementary Note 1) and $\Delta P_{Laplace}$ is the driving forces from the difference in Laplace pressure caused by the curvature gradient of cavity-microfiber (Supplementary Note 2).

Supplementary Note 4-Derivation of $L = 2m + \pi b$ for single knot from which the droplet detached.

As shown in Fig. 3e, the TCL is composed of two lines and a half-ellipse. The perimeter of an ellipse is equal to $2\pi b + 4(a - b)$. Therefore, the perimeter of a half-ellipse is equal to $\frac{1}{2} \times [2\pi b + 4(a - b)] + 2b$. The length of two lines is equal to $2(m - a)$. Therefore, the length of TCL can be written for a single knot from which the water droplet detached [13]:

$$L = \{\frac{1}{2} \times [2\pi b + 4(a - b)] + 2b\} + 2(m - a) = 2m + \pi b$$

where L is the length of TCL for a single knot from which the water droplet detached, m is the contact length between the fiber and water droplet, b is the minor semi-axis of the spindle knot and a is the major semi-axis of the spindle knot.

Supplementary Note 5-Maximum volume of water droplet collected by single cavity-microfiber.

We can evaluate the maximum volume of water droplet collected by single cavity-microfiber in Supplementary Fig.16:

$$V_M = \frac{\gamma \cos\theta L}{\rho g} = \frac{2\gamma\cos\theta}{\rho g}[(N-1)M + b(N\pi - \pi - 2N + 4)]$$

where γ is the surface tension of water ($\gamma \approx 7.2\times10^{-2}$ N m^{-1} at 25 ℃); ρ is the density of water (ρ=1 g cm^{-3}); g is the gravitational acceleration ($g \approx 10$ N kg^{-1}); θ is the contact angle of the water droplet on the fiber (θ=65.52°, cos65.52° ≈ 0.41437558099328, Supplementary Fig.16b); N is the number of identical neighboring knots for hanging the water droplet (N=2); b is the minor semi-axis of these spindle knots ($2b = 0.304$mm) and M is the contact length between the fiber and water droplet ($M = 1.223$mm).

Therefore, the maximum volume of water droplet collected by single cavity-microfiber in Supplementary Fig.16 is:

$$\begin{aligned} V_M &= \frac{2\times(7.2\times10^{-2}/1000)\times\cos 65.52^\circ}{(1/1000)\times(10/1000)}[(2-1)\times1.223 + (0.304\div2)\times(2\pi-\pi-2\times2+4)] \\ &\approx 2\times7.2\times0.0.41437558099328\times(1.223+0.152\times3.14) \\ &\approx 10.1456(\text{mm}^3) = 10.1456(\mu\text{L}) \end{aligned}$$

This agrees with the experimental data 10.13 μLvery well.

Supplementary Note 6-Driving force of water droplet on the intersectional cavity-microfibers.

Due to almost no difference of the solid-liquid interfacial tension on the two ends of water droplet, the driving force only arising from the difference of liquid-gas interfacial tensions on the two ends of water droplet and making coalescing water droplets transported to the intersection (Supplementary Fig.21) can be expressed as[14]:

$$F = 2\sigma_{\mathrm{lg}} l[\cos(\theta - \alpha) - \cos(\theta + \alpha)] \tag{5}$$

Where σ_{lg} is the liquid-gas interfacial tension of water droplet, l is the length of the contact line of water droplet inside the dehydrated cavity-microfiber on one dehydrated cavity-microfiber, α is half of the angle of intersectional structure of dehydrated cavity-microfibers, θ is the contact angle of water drop on the dehydrated cavity-microfiber. Obviously, the direction of resultant force is pointing towards the intersection (Supplementary Fig.21), resulting in that water droplet is propelled to move to the center of intersectional dehydrated cavity-microfibers.

Supplementary References

1. Bai, H., Ju, J., Sun, R., Chen, Y., Zheng, Y. M., Jiang, L. Controlled fabrication and water collection ability of bioinspired artificial spider silks. *Adv. Mater.* **23**, 3708–3711 (2011).
2. Tian, X. L., Chen, Y., Zheng Y. M., Bai, H., Jiang, L. Controlling water capture of bioinspired fibers with hump structures. *Adv. Mater.* **23**, 5486–5491(2011).
3. Ju, J., *et al.* A multi-structural and multi-functional integrated fog collection system in cactus. *Nat. Commun.* **3**, 1247 (2012).
4. Wang, S. J., Feng, S. L., Hou, Y. P., Zheng. Y. M. Controlling of water collection ability by an elasticity-regulated bioinspired fiber. *Macromol. Rapid. Comm.* **36**, 459−464 (2015).
5. Zheng, Y., *et al.* Directional water collection on wetted spider silk. *Nature* **463**, 640-643 (2010).
6. Hou, Y. P., Chen, Y., Xue, Y., Wang, L., Zheng, Y. M., Jiang, L. Stronger water hanging ability and higher water collection efficiency of bioinspired fiber with multi-gradient and multi-scale spindle knots. *Soft Matter* **8**, 11236–11239 (2012).
7. Wenzel, R.N. Resistance of solid surface to wetting by water. *Ind. Eng. Chem. Res.* **28**, 988-994 (1936).
8. Feng, S. L., Hou, Y. P., Xue, Y., Gao, L. C., Jiang, L., Zheng, Y. M. Photo-controlled water gathering on bio-inspired fibers. *Soft Matter* **9**, 9294–9297 (2013).
9. Daniel, S., Chaudhury, M. K., Chen, J. C. Fast drop movements resulting from the phase change on a gradient surface. *Science* **291**, 633-636 (2001).
10. Chaudhury, M. K., Whitesides, G. M., Yang, J.-T. How to make water run uphill. *Science* **256**, 1539-1541 (1992).
11. Lorenceau, E ́., Que ́re ́, D. Drops on a conical wire. *J. Fluid Mech.* **510**, 29-45 (2004).
12. Du, M., Zhao, Y., Tian, Y., Li, K., Jiang, L. Electrospun multiscale structured membrane for efficient water collection and directional transport. *Small* **12**, 1000–1005 (2016).
13. Hou, Y. P., Chen, Y., Xue, Y., Zheng, Y. M., Jiang, L. Water collection behavior and hanging ability of bioinspired fiber. *Langmuir* **28**, 4737−4743 (2012).
14. Dong, H. *et al.* Highly efficient fog collection unit by integrating artificial spider silks. *Adv. Mater. Interfaces* **3**,1500831 (2016).
