# Supplementary material for: Large-scale water collection of bioinspired cavity-microfibers
Source: Nat Commun. 2017 Oct 20;8:1080. doi: 10.1038/s41467-017-01157-4 (PMC5714965; doi:10.1038/s41467-017-01157-4)
Supplement: Supplementary file 2 — Description of Additional Supplementary Files [file 41467_2017_1157_MOESM2_ESM.pdf]

Description of Additional Supplementary Files

File Name: Supplementary Movie 1
Description: Tiny droplets move from the joint to the knot. Due to the surface energy gradient and Laplace pressure, the tiny water droplets can move directionally from the joint part to the knot part to form larger water droplet.

File Name: Supplementary Movie 2
Description: Droplets move up to the domain knot. The water droplets can overcome the gravity and move up to domain knots.
